# Using mounting, orientation, and design to improve bat box thermodynamics in a northern temperate environment

**DOI:** 10.1038/s41598-021-87327-3

**Published:** 2021-04-08

**Authors:** Amélie Fontaine, Anouk Simard, Bryan Dubois, Julien Dutel, Kyle H. Elliott

**Affiliations:** 1 Department of Natural Resource Sciences, McGill University, Ste-Anne-de-Bellevue, H9X 2E3 Canada; 2 Quebec Centre for Biodiversity Science, Montréal, H3A 1B1 Canada; 3 Ministère de la Forêt, de la Faune et des Parcs, Québec city, G1S 2L2 Canada; 4 CCM2 Architectes, Lévis, G6V 3X3 Canada; 5 Transition Énergétique, Québec city, G2K 0G9 Canada

**Keywords:** Bioenergetics, Conservation biology

## Abstract

Wildlife managers design artificial structures, such as bird houses and bat boxes, to provide alternative nesting and roosting sites that aid wildlife conservation. However, artificial structures for wildlife may not be equally efficient at all sites due to varying climate or habitat characteristics influencing thermal properties. For example, bat boxes are a popular measure employed to provide compensatory or supplementary roost sites for bats and educate the public. Yet, bat boxes are often thermally unstable or too cold to fulfill reproductive females needs in northern temperate environments. To help improve the thermodynamics of bat boxes, we tested the effect of (1) three mountings, (2) four orientations, and (3) twelve bat box designs on the internal temperature of bat boxes. We recorded temperatures in bat boxes across a climate gradient at seven sites in Quebec, Canada. Bat boxes mounted on buildings had warmer microclimates at night than those on poles and those facing east warmed sooner in the morning than those facing west or south. Our best new model based on passive solar architecture (Ncube PH1) increased the time in the optimal temperature range (22–40 °C) of targeted species by up to 13% compared to the most commonly used model (Classic 4-chamber) when mounted on a building with an east orientation (other designs presented in the Supplementary Information). Based on bioenergetic models, we estimated that bats saved up to 8% of their daily energy using the Ncube PH1 compared to the Classic 4-chamber when mounted on a building with an east orientation. We demonstrate that the use of energy-saving concepts from architecture can improve the thermal performance of bat boxes and potentially other wildlife structures as well.

## Introduction

Artificial dens, roosts, or nests for wildlife can help mitigate threats to animal conservation associated with habitat loss, climate change and direct human disturbance. Artificial roosts can be used to supplement available habitat, compensate for habitat lost, provide sites used during the nesting or gestation period, protect prey from predators, or shelter species from adverse weather^1–5^. Apart from direct conservation, artificial roosts can also facilitate the study or monitoring of wildlife populations^1^, as well as enhance citizen education and stimulate public involvement toward conservation^6^. Many investigators have reported the occupancy rates of artificial roosts, but fewer have explicitly studied the underlying causes influencing the selection of some models over others^7–9^. To avoid artificial roosts being used despite being suboptimal for successful reproduction or becoming periodically unsuitable with worldwide increasing extreme weather events^10–12^, it is important to understand how design, location, and mounting influence the roost microclimate, and ultimately, the preference and health of animals^13–17^. For example, the same box used on sites with different environmental conditions can unequally affect the energy budget of individuals, especially at critical life cycle stages, such as migration or reproduction^16–20^.

Wildlife living in temperate zones must adapt to a wide range of temperatures, which can vary considerably throughout the year. For instance, in Quebec, Canada, while the average summer high temperature ranges from 25 to 30 °C, temperatures during the night are sometimes under 0 °C. For insectivorous bats and birds, the negative effect of ambient temperature on food availability compounds this problem^21^. Torpor, a state of decreased physiological activity including low body temperature and metabolic rate, is one strategy used by insectivorous bats to cope with the costs of maintaining a high body temperature^22,23^. Although beneficial to synchronize birth to the peak of resources^24^ or allow bats to save energy on cool days when insects are not available, entering torpor during summer may also delay female reproduction by slowing fetal development and reducing milk production^25–27^, with negative consequences on the probability of survival throughout hibernation for both mother and offspring^22,23,28,29^. To reduce torpor use and optimize their fitness, female little brown bats (*Myotis lucifugus*) inhabit warm roosts close to feeding sites (home range within 20–30 ha ^30^). The thermoneutral zone of little brown bats is 32–37 °C^31^, meaning that an individual can maintain internal temperatures with minimal metabolic regulation within this range. For bats, it is also meaningful to determine at which temperature, although outside of that ideal range, females can manage to maintain adequate temperatures without entering torpor or suffering from detrimental effects. When roost temperature falls below 20 °C in gestation, or below 22 °C in lactation, females little brown bat use torpor 50–70% of the time^32^. On the other hand, when temperature reaches 40 °C, individuals exhibit behavioral thermoregulation, moving from upper to lower areas of houses’ attics, most likely to avoid the detrimental effects of overheating (e.g. dehydration, heat stroke, or death)^14,32^. Although not functionally equivalent to the thermoneutral zone, a temperature range of 22–40 °C is likely suitable for breeding females to remain homeothermic without altering their behavior, therefore minimizing energy expenditure while maximizing reproductive success^18–20,32^.

North American insectivorous bats are important pest consumers, yet this ecological and economical services are becoming scarcer, as more than half of North American insectivorous bat species are declining due to anthropogenic pressures, exacerbated more recently by exposure to White-Nose Syndrome (WNS)^34,35^. Bats have long life expectancy and reproduce slowly, which makes population recovery slow^36^. For example, the little brown bat can live up to 30 years in the wild and females give birth to only a single pup per year^37^. As bats spend over half of their lives within roosts^38^, providing suitable roost sites can aid population recovery for species that are declining^20^. Experiments have occurred since the 1980s to test bat boxes effectiveness as conservation measures. The bat box’s microclimate, especially the internal temperature, is one of the most important selection criteria for bats^39–43^. The internal temperature is influenced by many factors, including bat box orientation, mounting, sun exposure, colour, design, construction material, and the number of occupants^15,16,41,42^. Proximity to water, species-specific habitat type, proximity to an existing roost site, time since installation and clustering of bat boxes also increase the probability of bat box colonization^7,42^. Colonization success also rises when using multiple narrow chambers that allow bats to roost side-by-side and employing an open bottom design that reduces colonization risk of non-target species (e.g. birds, small mammals, wasps) and feces accumulation^7^. Finally, height above ground, form and size of the bat box can also influence the colonization success but vary widely among roost types and species, with vespertilionids roosting in tree cavities and buildings most commonly using bat boxes^7,44^.

Despite attempts by conservationists to address the causes of variability listed previously, the colonization rate of bat boxes varies widely^7,16,44–46,58^, often with low rates in urban or suburban northern temperate environments (e.g. occupancy rate of 1–48% in Canada, Northern United States, Poland, and Englan^44,47,48^; but see^49^). Most studies on bat box suitability occurred in temperate and warm climates, such as in the Central and Southern United States and the Mediterranean region. Few researchers have adapted bat boxes to northern temperate conditions, where the average temperature in June is equal or less than 20 °C^41,49,50^. Knowledge gained during the last few decades on passive solar architecture, used to heat or cool residential houses, could be transposed to bat boxes (and other forms of wildlife artificial roost development) to improve their thermodynamics and be suitable for a colder and larger range of climatic conditions^51–53^. Passive solar designs take advantage of a building’s geographic location and climatic conditions, building’s shape, orientation, construction materials, openings, and more to minimize energy use for heating and cooling to maintain thermal conditions that are suitable for its inhabitants^51^. These design principles reduce heating and cooling loads through energy-efficient strategies, such as using thermal masses to store the heat, which are redistributed through radiation at night^52^.

The present study aimed to improve the thermal properties of artificial bat boxes to better meet the thermal preferences of reproductive female little brown bats living in Quebec, as estimated via the extended optimal temperature range of 22–40 °C (EOTR). First, we tested the impact of three mountings (poles, non-heated buildings, and heated buildings) and four orientations (south, east, and west for buildings and south, east, and south-east for poles) on the thermodynamics of the most commonly used bat box model, the Classic 4-chamber. We then evaluated the impact of bat box design on its thermodynamic by testing four commonly used bat box models and eight newly designed models (Supplementary Table S1). As newly designed models were improved over the years, we only present results comparing the thermodynamics of our best newly designed model, the Ncube PH1, to the most commonly used model, the Classic 4-chamber in the main manuscript. Finally, we modelled daily energy expenditure of gestating and lactating little brown bats using these two boxes. We predicted that external factors, such as an east-facing orientation and mounting on a heated building, could improve thermal properties of bat boxes and favour time spent in the EOTR of 22–40 °C of reproductive female little brown bats. We also expected that integrating concepts of energy saving and passive heating into bat box designs would improve the thermal properties and increase the amount of time within an EOTR of 22–40 °C compared to traditional designs. Finally, we expected that thermal improvements to bat boxes, as measured during our field experiments, should lower the modelled daily energy expenditure of reproductive female little brown bats. We expected bats to save energy especially at night and in the early morning, when reproductive females are thermally challenged as they rewarm after nightly torpor bouts.

## Results

### Mounting

**Figure 1:**
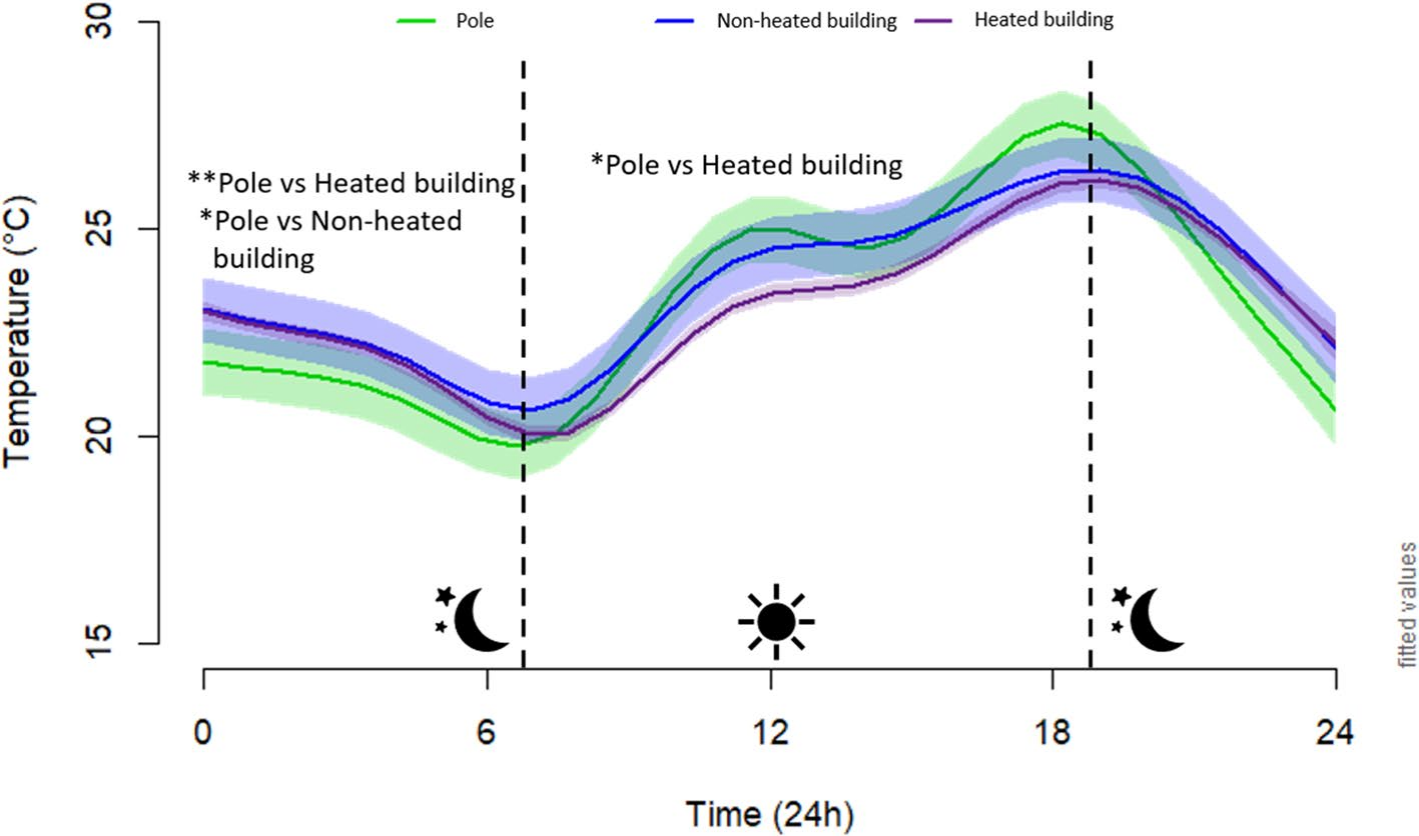
Estimated hourly patterns for the different Classic bat box mountings in Quebec, in 2017 (pole: n = 6, non-heated building: n = 6, and heated building: n = 6). The estimated values are based on a generalized additive mixed model, accounting for time, date, orientation, external temperature, site, and individual bat box identity. Values of fixed factors have been set to: date = July 6, orientation = east, external temperature = 18 °C. The asterisks (*) represent significant differences between structures during the day and night. P = pole and B = building.

During 122 days, from mid-May to mid-September 2017, we recorded 2928 temperature data points for each of the 18 Classic 4-chamber bat boxes, which were either mounted on poles (n = 6), non-heated buildings (n = 6), or heated buildings (n = 6) at two sites in Quebec, Canada. Based on a generalized additive mixed model, we estimated that average daily temperatures of Classic bat boxes varied among mounting types, sites, and with time of day (Fig. 1, Supplementary Tables S2 and S3). At night, the Classic bat boxes average temperature was between 1 and 1.5 °C warmer when mounted on heated or non-heated buildings than poles while the opposite occurred during the day (Fig. 1). The percentage of time below, between, and above the EOTR were relatively similar among mounting type. Temperature varied from 6.5 to 44 °C in Classic bat boxes on poles, from 6.5 to 48.5 °C in Classic bat boxes on non-heated buildings, and from 7 to 49 °C in Classic bat boxes on heated buildings. The minimal and maximal temperature of Classic bat boxes were generally similar among mountings at the same site, varying from 0.5 to 5 °C (Table 1).

**Table 1:** Percentage of time below, between, and above the extended optimal temperature range of 22 − 40 °C, and minimal and maximal temperature inside and outside of Classic bat boxes from mid-May to mid-September 2017 at intermediate and warmer sites in Quebec, Canada presented per mountings and orientations.

Orientation	Measures	Intermediate 1 site			Warmer 2 site		
		Heated buiding	Non-heated buidling	Pole	Heated buiding	Non-heated buidling	Pole
East	Time below 22 °C (%)	59	54	53	50	47	55
	Time between 22 and 40 °C (%)	41	46	47	47	52	45
	Time above 40 °C (%)	0	0	0	3	1	0
	T_min_	7	7.5	7	8	7.5	6.5
	T_max_	38	38.5	39	49	45	43.5
South-East	Time below 22 °C (%)			59			54
	Time between 22 and 40 °C (%)			41			45
	Time above 40 °C (%)			0			1
	T_min_			6.5			7
	T_max_			35			44
South	Time below 22 °C (%)	62	58	55	47	53	55
	Time between 22 and 40 °C (%)	38	42	45	53	47	45
	Time above 40 °C (%)	0	0	0	0	0	0
	T_min_	7	7.5	7	9.5	8	6.5
	T_max_	40	38	38	40	41	41
West	Time below 22 °C (%)	61	59		52	52	
	Time between 22 and 40 °C (%)	39	40		45	46	
	Time above 40 °C (%)	0	1		3	2	
	T_min_	7	6.5		8.5	7.5	
	T_max_	39	41.5		49	48.5	

### Orientation

**Figure 2:**
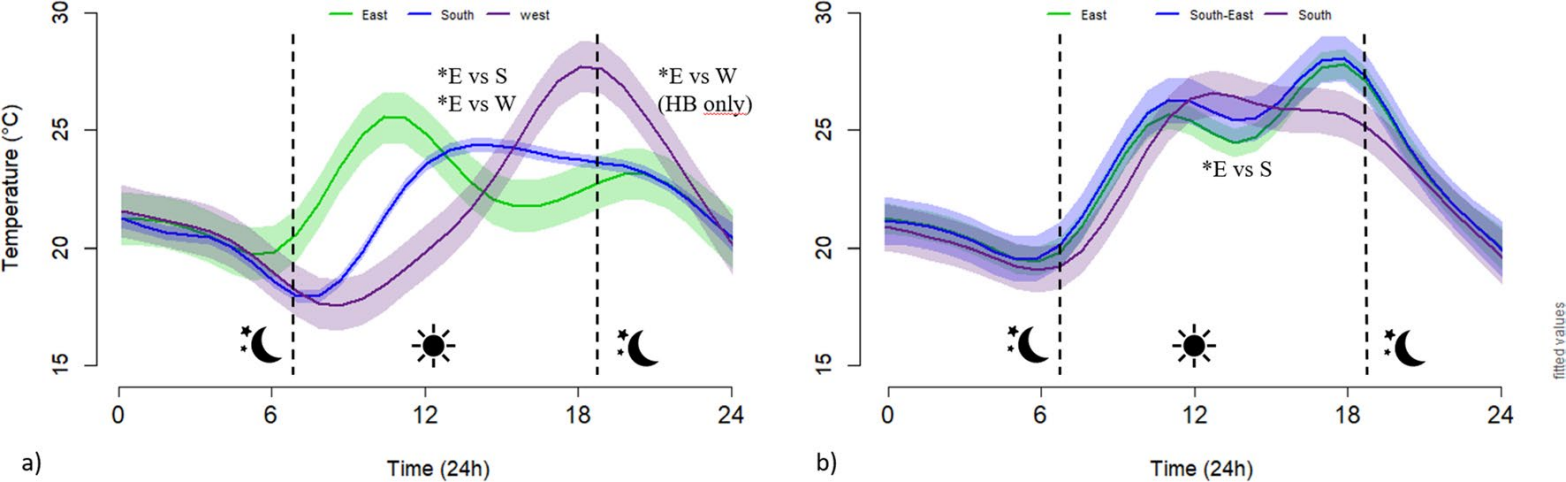
Estimated hourly patterns for the different bat box orientations in Quebec, in 2017. Bat boxes facing east (n = 4), south (n = 4), and west (n = 4) on buildings (**a**), and facing east (n = 2), south (n = 2) and south-east (n = 2) on poles (**b**). The estimated values are based on a generalized additive mixed model, accounting for time, date, external temperature, structure, site, and individual bat box identity. Values of fixed factors have been set to: date = July 6, external temperature = 18 °C. The asterisks (*) represent significant differences between orientations during the day and night. E = east, S = south, W = west, and SE = south-east. HB = heated building.

During 122 days, from mid-May to mid-September 2017, we recorded 2928 temperature data points for each of the 18 Classic bat boxes, which were either facing east (n = 4), south (n = 4), and west (n = 4) on buildings, or facing east (n = 2), south (n = 2) and south-east (n = 2) on poles, at two sites in Quebec, Canada. Based on a generalized additive mixed model, we estimated that average daily temperatures of Classic bat boxes varied among orientations, mounting types, sites, and with time of day (Fig. 2, Supplementary Tables S4 and S5). On buildings, west-facing Classic bat boxes were significantly warmer in the early evening and colder in the early morning than those facing east. During the day, temperatures in east-facing Classic bat boxes were significantly different than those facing south or west, being warmer especially in the morning (Fig. 2). On poles, the only significant difference was at mid day, when south-facing Classic boxes were warmer than those facing east. The percentage of time below, between, and above the EOTR varied among orientations. The highest percentage of time in between the EOTR occurred for Classic bat boxes with an easterly orientation, while the highest percentage of time above the EOTR occurred for bat boxes with an east and west orientation at the warmer site. Temperature varied from 6.5 to 49 °C in Classic bat boxes facing east, from 6.5 to 44 °C in Classic bat boxes facing south-east, 6.5–41 °C in Classic bat boxes facing south, and from 6.5 to 49 °C in Classic bat boxes facing west. The minimal and maximal temperature of Classic bat boxes were generally similar, among orientations at the same site, varying from 0.5 to 5 °C (Table 1).

### Design

**Figure 3:**
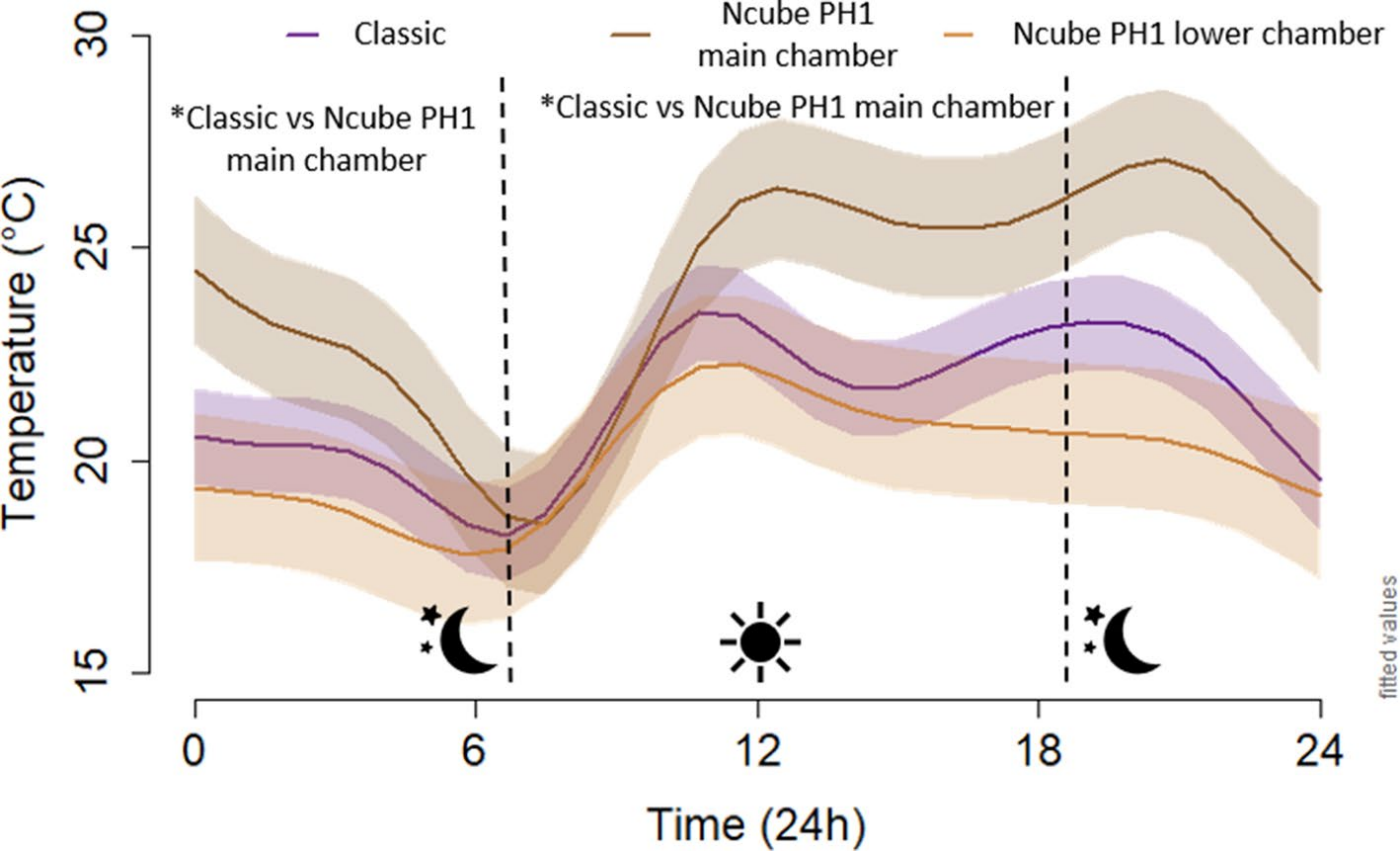
Estimated hourly patterns for the Classic (n = 11) and the Ncube PH1 models (main and lower chambers, n = 8). The estimated values are based on a generalized additive mixed model accounting for time, week, year, structure, external temperature, site, and individual bat box identity. Values of fixed factors have been set to: week = first half of July, year = 2019, structure = building, external temperature = 18 °C. The asterisks (*) represent significant differences between models during the day and night.

From mid-May to mid-September 2016–2019, we recorded 2928 temperature data points per year and per bat box at seven sites in Quebec, Canada. We tested in total 12 bat box designs mounted on poles and buildings facing east, but only present results comparing the thermodynamics of our best newly designed model, the Ncube PH1, which included a main and a lower chamber (n = 8), to the Classic 4-chamber (n = 11). Based on a generalized additive mixed model, we estimated that average daily temperatures varied between the Classic and the main chamber of the Ncube PH1 model, (Fig. 3, Supplementary Tables S6 and S7, see Supplementary Fig. S1 for all models). Temperatures in the Classic and Ncube PH1 were significantly warmer than the outside temperature during both night and day. The main chamber of the Ncube PH1 was significantly warmer than the Classic during both night and day, being on average 3.5 ± 1.5 °C warmer during the night, 3 ± 1.5 °C warmer in afternoon, and similar in the morning from 700 to 1100.

Bat boxes were installed at seven sites separated into cooler (n = 2, T_*x̅* in June_ = 11 °C), intermediate (n = 3, T_*x̅* in June_ = 16 °C), and warmer sites (n = 2, T_*x̅* in June_ = 19 °C). For comparative purposes, we present the percentage of time below, in between, and above the EOTR for bat boxes on buildings at intermediate sites only; results at warmer and cooler sites being similar with a higher and lower mean temperature respectively (Table 2, see Supplementary Fig. S2 for all models). The Classic was in the EOTR 46% of the time and above the EOTR 2% of the time. Considering the bats can use the lower chamber when above 40 °C in the main chamber, the Ncube PH1 was in the EOTR 58% of the time and never above the EOTR, therefore increasing by 12% the amount of time in the EOTR compared to the Classic.

**Table 2:** Percentage of time below, between, and above the extended optimal temperature range of 22–40 °C, and minimal and maximal temperature in Classic bat boxes, Ncube PH1 bat boxes, and outside (external temperature) from mid-May to mid-September 2019 in Quebec, presented per climatic and mounting types.

Models	Measures	Building			Pole		
		Cooler	Intermediate	Warmer	Cooler	Intermediate	Warmer
Classic	Time below 22 °C (%)	72	52	36	79	53	45
	Time between 22 and 40 °C (%)	28	46	60	21	46	51
	Time above 40 °C (%)	0	2	4	0	1	4
	T_min_	− 4.5	2	7	− 1	2	5
	T_max_	45.5	48	55	31	43.5	47.5
Ncube PH1: main chamber	Time below 22 °C (%)	59	42	32	51	47	42
	Time between 22 and 40 °C (%)	39	54	58	49	53	57
	Time above 40 °C (%)	2	4	10	0	0	1
	T_min_	− 3	1.5	7	0	4.5	6.5
	T_max_	47	49	53	34.5	38	47
Ncube PH1: lower chamber	Time below 22 °C (%)	77	64	51	79	61	50
	Time between 22 and 40 °C (%)	23	36	49	21	39	50
	Time above 40 °C (%)	0	0	0	0	0	0
	T_min_	− 4.5	2.5	6	0	4	7
	T_max_	38.5	40	41	31.5	43.5	43.5
External	Time below 22 °C (%)	92	74	63	92	74	63
	Time between 22 and 40 °C (%)	8	26	37	8	26	37
	Time above 40 °C (%)	0	0	0	0	0	0
	T_min_	− 5	− 2	5	− 3	− 2	5
	T_max_	29.5	31.5	42.5	28.5	31.5	42.5

**Figure 4:**
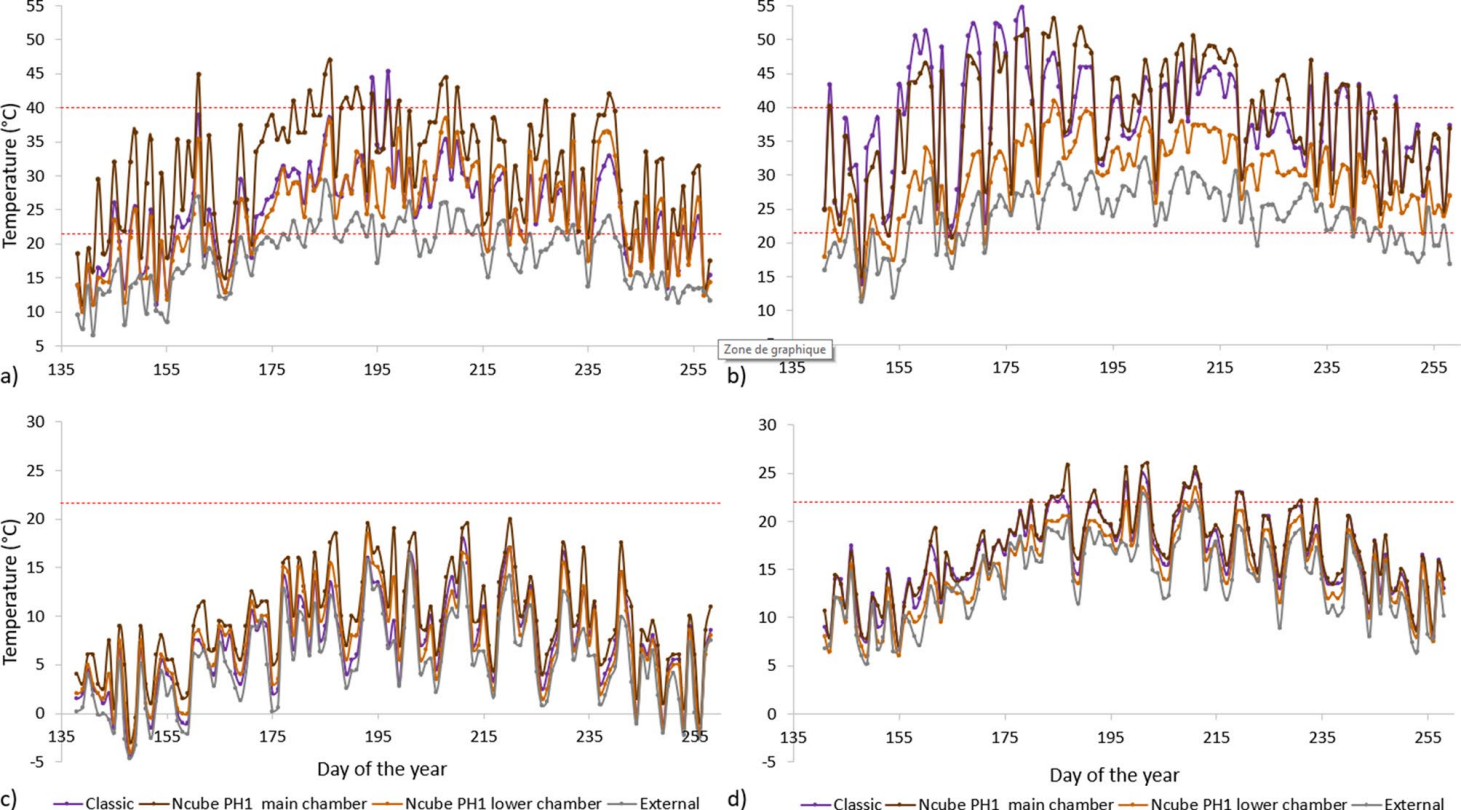
Daily minimum and maximum temperatures in the Classic and the Ncube PH1 bat boxes on buildings and the external temperature in 2019 at cooler and warmer sites, in Quebec. (**a**) daily maximum temperatures at the cooler site, (**b**) daily maximum temperatures at the warmer site, (**c**) daily minimum temperatures at the cooler site, (**d**) daily minimum temperatures at the warmer site. The red dotted lines represent the extended optimal temperature range of 22–40 °C.

The minimal temperature of the Classic and the Ncube PH1 were similar (2 °C and 1.5 °C respectively) but the maximal temperature differed given the use of the lower chamber of the Ncube PH1 (48 °C and 40 °C respectively; Table 2). From mid-May to mid-September 2019, daily minimum temperatures recorded in the Classic were slightly warmer than external temperatures, but slightly colder than the Ncube PH1 main chamber at both cooler 1 and warmer 2 sites (Fig. 4). Daily maximum temperatures in the Classic were warmer than external temperatures, similar to the Ncube PH1 lower chamber, and colder than the Ncube PH1 main chamber at the site cooler 1. At the site warmer 2, daily maximum temperatures in the Classic were warmer than the Ncube PH1 lower chamber, but similar to the Ncube PH1 main chamber, both overheating frequently, with 50 and 54 days with daily maximum temperatures over 40 °C respectively (Fig. 4). Overheating events occurred only once at warmer sites for the Ncube PH1 lower chamber on buildings, which spent < 1% of the time above 40 °C (one two hours long overheating bout; Table 2). Overheating events occurred at warmer and intermediate sites for the Classic model, which spent 4% of the time above 40 °C at warmer sites (52 1–6 h long overheating bouts in 2019) and 2% at intermediate sites (12 1–6 h long overheating bouts in 2019).

### Bioenergetic modeling

**Figure 5:**
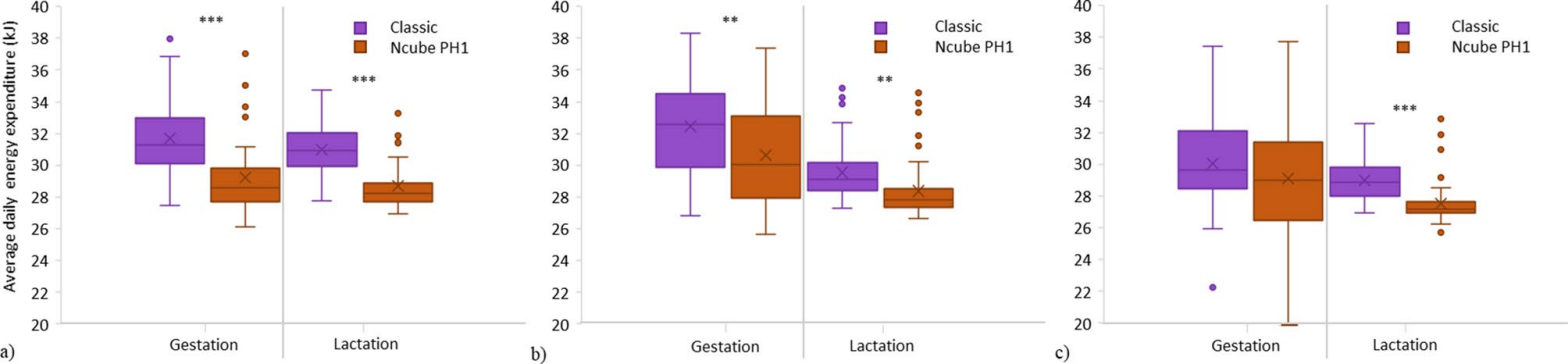
Average daily thermoregulatory energy expenditure (in kilojoules) from bioenergetic modeling for a female bat during the gestation and lactation period in a Classic versus Ncube PH1 bat box on building at: (**a**) cooler, (**b**) intermediate, and (**c**) warmer sites in Quebec, in 2019.

We estimated average daily thermoregulatory energy expenditures using bioenergetic modeling for a female little brown bat during the gestation and lactation period based on internal temperatures recorded in 2019 in a Classic 4-chamber versus a Ncube PH1 bat box on building at cooler, intermediate, and warmer sites in Quebec, Canada. We selected the internal temperature of the Ncube PH1 main chamber when equal or lower than 40 °C and the lower chamber when above 40 °C. The internal temperature of the Classic was based on the middle chamber (chamber 3 from the front) at all times (see the methods for more details). Predicted average daily energy expenditure was reduced by 3–8% during gestation in the Ncube PH1 compared to the Classic bat box model (Fig. 5). During lactation, energy savings varied between 5 and 7%. Energy saving differences were higher in cooler sites compared to warmer sites but were significant at all sites and reproductive periods, except during gestation at warmer sites.

## Discussion

To be a valuable conservation measure, artificial roosts must be adapted to the species’ needs and location^54^. At our study sites, an easterly orientation and mounting on a building improved the thermodynamics of bat boxes: it increased temperature at night and in the morning and provided a better thermal stability, likely favouring the reproductive success of little brown bats. Although it is impossible to always be in the EOTR with concepts of passive heating and heat conservation in a northern temperate environment, those concepts improved the thermodynamics of our newly designed Ncube PH1 model over the Classic model by increasing the amount of time within EOTR by 7–13% and remaining on average 4.5 °C warmer at night. Finally, we confirmed that thermal improvements of bat boxes could minimize the energetic costs and decrease daily energy expenditure for reproductive females little brown bats who occupy them in a northern temperature environment. Average daily energy expenditure differences were greater during lactation than gestation and at cooler than warmer sites, with an average saving up to 8%.

### Mounting

Although well covered for birds, few studies have evaluated the effect of mountings on roost use for bats^44,55^. A review by^44^ stated that bat boxes are placed on a variety of structures, most commonly on trees, poles, and buildings, but that bat boxes had highest occupancy rates or are most frequently used when mounted on buildings and poles than on trees^33,48,56–58^. However, none of these studies evaluated how mounting type influences the thermodynamics of the box. On our study sites, mounting on buildings, heated or not, resulted in warmer bat box internal temperatures than on poles during the night with the reverse during the day. The temperature variance was slightly higher for bat boxes on poles than those on buildings. This can be explained by a longer solar exposure (from sunrise to sunset) for bat boxes installed on poles than buildings, as the latter were shaded by the building walls for part of the day. Buildings protect bat boxes from the wind coming from behind and partially from the wind coming from the sides. Buildings also enhance heat retention, providing more stable conditions than those on poles in our study area. We detected no difference between heated and unheated buildings, day or night. The non-heated buildings tested in our study were brick or insulated buildings, so perhaps if we had tested non-insulated buildings, that have a lower heat retention capacity, such as old barns or sheds, we would have obtained different results.

### Orientation

Several studies report that the orientation of bird and bat boxes influences occupancy rates, with south or east-facing boxes being favoured in a northern temperate environment^58–61^. Few investigators have explicitly examined whether nest orientation preference appeared correlated with bird nest temperatures^61–63^. For little brown bats established maternity colonies in artificial roosts that received ≥ 7 h of sunlight in Pennsylvania^8^. In Australia, the effect of colour on box temperatures was influenced by a range of factors, including orientation. They found that the box maximum daytime temperature, and the difference between the box and the ambient maximum daytime temperature, were most pronounced for bat boxes facing north and west, the orientations that receive the greatest amount of solar radiation during the hottest period of the day^16^. Also in Australia, a study reporting bat box orientation preferences by Gould’s wattled bats (*Chalinolobus gouldii*) showed that orientation influenced box temperature, with west-facing boxes being coolest^64^.

In our study, the effect of bat box orientation on internal temperature depended on the mounting type, the influence being stronger on buildings than on poles. On buildings, bat boxes facing east warmed sooner in early morning. Moreover, an easterly orientation enhanced the time in the optimal temperature by up to 6% compared to a westerly orientation at the intermediate site. Estimated temperatures for south-facing boxes on buildings were surprisingly lower or equal to those estimated for east or west-facing boxes. Our south-facing boxes should have benefited from longer solar exposure during the warmest period of the day and should have recorded higher temperatures than bat boxes facing east or west; others did find that black-coloured and south-facing boxes recorded the highest temperatures^15^. We suspect that roof eaves facing south and north created shade for a significant amount of time. Orientations tested in this study had little influence on bat box temperature when mounted on poles without differences in temperature increase in the mornings. At night, we suspect insufficient heat retention on poles, while during the day, one side is always fully exposed to the sun. We argue that an east-facing orientation is generally preferable in a northern temperate environment as in Quebec. An east-facing orientation, especially on buildings, maximizes the time in the EOTR, while also warming up sooner in the cooler early mornings.

### Design

Design is one of the most important characteristics determining attraction of bats or birds to structures, and design refinement improves the frequency use of nest and bat boxes^7,65,66^. Review^44^ identified more than 48 type of bat boxes varying in their materials, size, and shape. However, the authors noted that only a few studies explain how structural characteristics of bat roosts influence internal temperatures. In Portugal, temperatures of artificial roosts painted black (vs. white or gray) were most comparable to building roosts and had the highest use by bats^42^. In the USA, the “rocket” box, which was the largest roost, remained within the critical temperature thresholds the greatest proportion of time^67^. In Australia^68^, found that bat box colour, chamber sequence, construction materials, and vents influenced internal temperatures. In Canada, bats preferentially selected heated over non-heated bat boxes^20^.

Out of the 12 models we tested, including traditional and newly designed models, the Ncube PH1, improved thermal performance both on poles and buildings by increasing time in the EOTR and being warmer at night without overheating during the day. Such improved thermal performance is likely attributed to: (1) a passive heating zone that improves heat gain and retention of the bat box, (2) a thick insulation buffering against temperature fluctuations, (3) a reduced chicane entrance that decreases air exchange and heat loss, and (4) an additional cool chamber at the bottom, where bats can easily and safely go when the main chamber overheats. Still, at warmer sites, the Ncube PH1 on buildings spent a few hours above 40 °C on sunny days, when external temperatures exceeded 28 °C around noon. We suggest three different ways to reduce overheating of the Ncube PH1 at sites similar or warmer than our warmer sites depending on the specific environmental conditions of the site: (1) a modification of the passive heating zone, from fully juxtaposed with the main chamber to a halfway position to reduce heat diffusion, (2) a wider entrance to increase the air flow and heat loss, or (3) a lighter colour (green, brown, or grey) that minimises heat absorption^16^. Black has low reflectance and is commonly acknowledged as the best colour to use in northern regions (where the average high temperature in July is ≤ 29 °C) to increase the internal temperature^69^. However, black also increases the number of overheating events, even in temperate climates^15^. Therefore, we advise local testing to find what colour best fits local conditions.

Four criteria guided the elaboration of the newly designed models: (1) thermal preferences and requirements of little brown bats minimizing torpor use: EOTR of 22–40 °C^29^, (2) a passive heating design including a passing heating zone, a reduced opening, and insulation, (3) a relatively light weight, and (4) a low cost. Thanks to a transdisciplinary approach integrating knowledge related to biology/ecology (bat scientist) and material properties and thermodynamics (architects and engineers), we translated energy saving concepts from human eco-housing to bat boxes and created a versatile passive heating design well adapted to a wide range of northern temperate environments. The Ncube PH1 can easily be installed by two people (one person to hold the box and one person to fix the box in place) with a screwdriver and screws on a building or with U-bolts with plates and hex nuts on a pole. However, while prioritizing the optimization of the thermodynamics of the bat box, we failed to keep a low cost. Its price of ~ $800 CAD (including carpenter time fees) is around four times higher than a Classic 4-chamber and almost twice the price of a regular rocket bat box from local retailers. We still consider that such investment could be justifiable when applied as a compensation measure to roost exclusion or habitat destruction.

### Bioenergetic modeling

Roosts have the potential to influence energy expenditure considerably^70^ through thermoregulation and/or passive rewarming from daily torpor^71,72^. This is especially true for reproductive females that select warm roosts and use shorter and shallower torpor bouts compared to non-reproductive individuals to optimise pup growth during gestation and milk production during lactation^8,41,73^. Patterns of torpor use suggest that bats in buildings save more energy than rock-roosting individuals by roosting in the warmer microenvironments of buildings^4^. Energetic modeling assuming that bats re-warm from torpor passively as the internal temperature of the heated bat box rises to 32 °C in the morning, demonstrates major energy savings compared to active rewarming in regular roosts^20^. Although the warmer temperatures recorded in the morning in the Ncube PH1 did not allow passive rewarming, a warmer temperature at night and a quicker increase in the morning most likely results in shallower and less-frequent use of torpor and lower energy costs of rewarming after torpor bouts. Those energetic advantages should therefore favor earlier births and faster juvenile growth, which should increase the fitness of both mother and pup^4,74^.

Female bats using the Ncube PH1 2019 rather than the Classic model would save between 3.2 and 7.8%, with the highest saving at cooler sites, underlining the importance of efficient bat boxes adapted to a northern temperate environment. The Ncube PH1 would be likely beneficial in particular north of the 50° N, where suitable natural roost availability is scarce and basic bat boxes, similar to the Classic model, are frequently used as maternity colonies. Some parameters in our bioenergetic models, such as time in torpor and foraging flight time, are flexible and depend on weather, sex and reproductive status^75^. Furthermore, the number of bats in a box will also influence individual energy expenditure^76^. Nonetheless, our model estimates reflect the bioenergetic advantages of our newly designed model and fit those reported in the literature for little brown bats^20,77^.

## Conclusions

We recommend erecting bat boxes facing east and mounted on buildings when safe cohabitation with humans is possible. We also advise bat box designs that include insulation and a passive heating zone similar to the Ncube PH1 that buffers against suboptimal temperatures and increases the time in the EOTR (see Supplementary Figs. S3–S6 for the original design plan and batwatch.ca for the improved 2020 design plan). Insulated models could retain heat generated through social thermoregulation better than uninsulated designs, which should also be considered in future studies. Moreover, a cool open chamber at the bottom is a good addition to any bat box model in case of overheating events. We tested the Ncube PH1 model across a range of temperatures in Quebec and demonstrated its thermal advantage for female little brown bats in a northern temperate environment. Despite the better thermal suitability of the Ncube PH1 compared to the Classic model, offering several alternative to bats is still recommended. There is evidence that box deployment in clusters is important to accommodate individuals of different sexes and reproductive status throughout the whole season and to a larger range of meteorological conditions^7,75,80,81^. A cluster of different bat box models and/or orientations also provide opportunities for roost switching^78^ and social interactions^79^. Little brown bats roosting in natural habitats, have also been observed to prefer forest stands surrounded by a large number of snags allowing roost switching^82^.

Our Ncube PH1 better meets the little brown bat (federally listed as Endangered in Canada due to White-Nose Syndrome) requirements than most commonly used bat box models, such as the Classic 4-chamber, and can serve as an alternative roost for bats excluded from dwellings or to enhance high quality habitats where roost availability is limited^17^. Nonetheless, assuring an abundance of natural roosting sites remains desirable for bat conservation, especially for tree-roosting species. When installing bat boxes, we encourage local testing and careful consideration of the habitat, availability of suitable roosts in the environment, species present in the local bat assemblage, target species, structure and orientation, although the first two parameters are difficult to measure. Since this study showed the theoretical value of our newly designed bat box, the next step is to test its colonization success and its functionality for bats on a large scale. Artificial structure optimization using human architecture concepts shows a great potential to improve conservation tools for other taxa like birds or other hollow-dependent mammals.

## Materials and methods

### Mounting experiment

**Figure 6:**
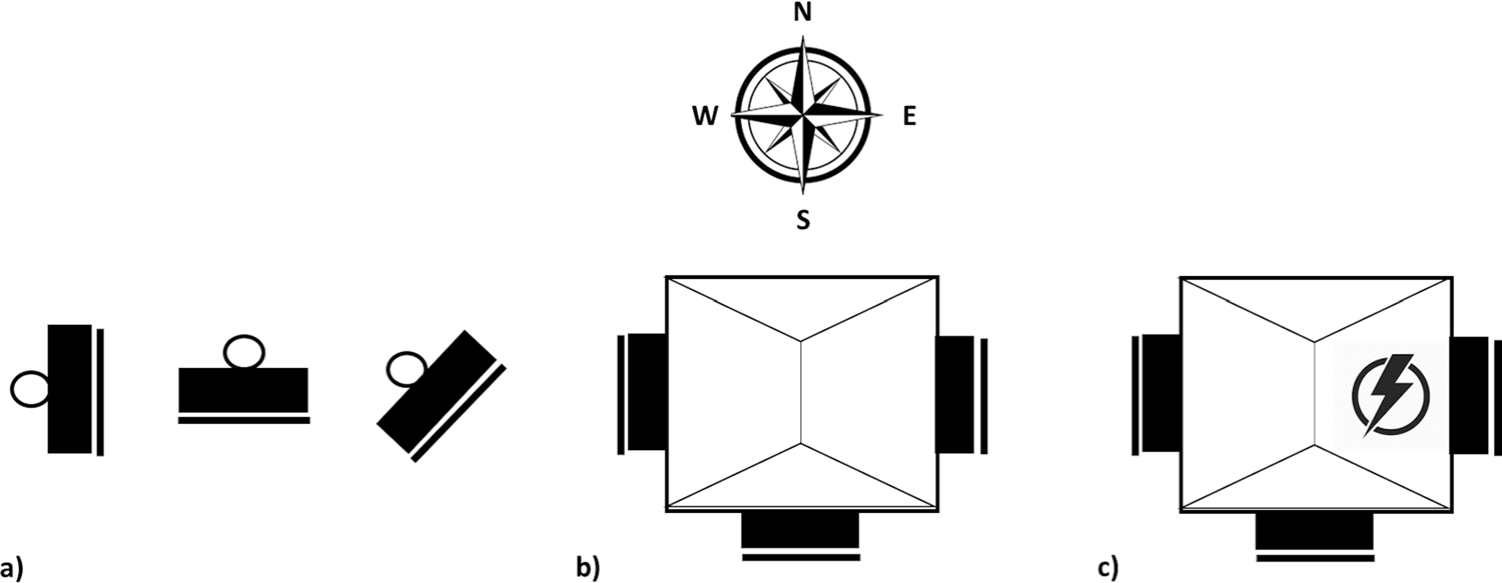
Schematic view of the experimental design for the orientation and mounting as tested for bat boxes on two sites in Quebec, in 2017 on (**a**) poles, (**b**) non-heated buildings and (**c**) heated buildings.

**Figure 7:**
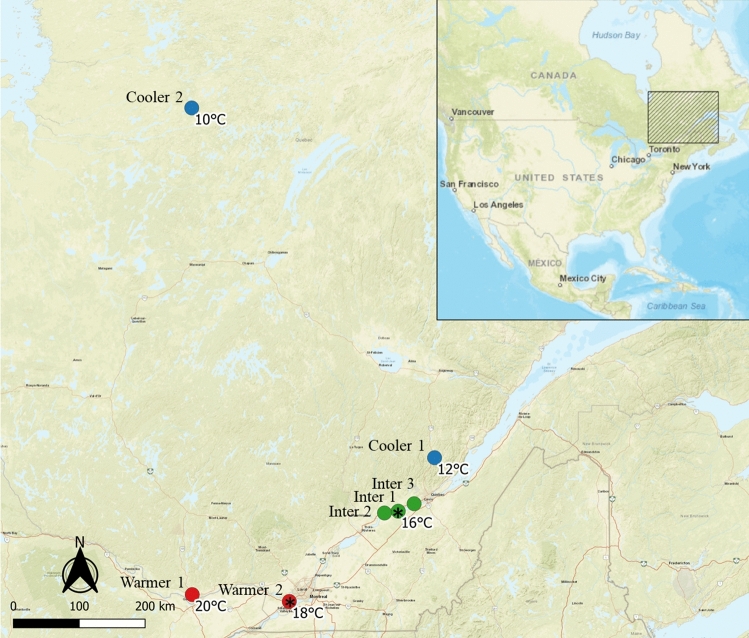
Location of the bat box installation sites at cooler (blue), intermediate (green) and warmer (red) sites in Quebec, Canada, from 2016 to 2019 with mean temperature in June. The Montmorency forest is considered a colder site due to its high elevation of 750 m. Structure and orientation tests occurred at sites represented by a star. Map derived from ESRI Standard https://server.arcgisonline.com, modified with QGIS 3.4.4. software (www.qgis.org).

For our first experiment, we tested the effect of mountings on bat box thermodynamics with 18 bat boxes mounted on (1) metal poles (diameter = 6 cm; n = 6), (2) non-heated buildings (n = 6), and (3) heated buildings (n = 6; Fig. 6) at two sites in Quebec, Canada (Fig. 7). We used the most common bat box model, the Classic 4-chamber (https://www.batcon.org/wp-content/uploads/2020/09/4-Chamber-Nursery-House-Plans.pdf; paint in black with no vents to be adapted to a northern temperate environment). We recorded bat box internal temperatures (T_int_) every hour using iButtons (https://www.ibuttonlink.com/products/ds1921g) from mid-May to mid-September 2017.

### Orientation experiment

For our second experiment, we tested the effect of orientations on bat box thermodynamics at the same two sites, using again 18 bat boxes facing east (n = 4), south (n = 4), and west (n = 4) on buildings, and facing east (n = 2), south (n = 2) and south-east (n = 2) on poles. We installed the same commonly used Classic 4-chamber bat box and recorded internal temperatures every hour (T_int_), from mid-May to mid-September 2017, using iButtons.

### Design experiment

Our third experiment aimed to test the effect of design on bat box thermodynamics using 12 models of bat boxes, mounted facing east on poles and buildings at seven sites (Fig. 7). As Quebec mean summer temperatures can vary up to 26 °C from south to north, we separated sites into cooler (n = 2, T_*x̅* in June_ = 11 °C), intermediate (n = 3, $${\text{T}}_{{\overline{x}\;{\text{in}}\;{\text{June}}}}$$ = 16 °C), and warmer sites (n = 2, T_*x̅* in June_ = 19 °C). The two warmer sites and two intermediate sites had pole and building mountings, the other sites had either pole or building mountings. We recorded bat box internal temperatures (T_int_) every hour using iButtons from mid-May to mid-September 2016–2019. The 12 bat box models tested included a Classic 4-chamber, an European fibrocement 1-chamber model, two Insulated rocket models (with and without a solar heated system), and eight new models based on passive solar concepts (Supplementary Table S1). Four criteria guided the elaboration of the newly designed models: (1) thermal preferences and requirements of the endangered little brown bat minimizing torpor use: EOTR of 22–40 °C^32^, (2) a passive heating design including a passing heating zone, a reduced opening, and insulation, (3) a relatively light weight, and (4) a low cost (less than $500 CAD). The last two criteria were selected to facilitate large-scale implementation of an improved model assuming its success at the first criterion. We painted all bat boxes were black to maximise solar radiation absorbance^16^. The newly designed bat box was conceptualized based on thermodynamics principles used in solar passive architecture, such as heat transfer (radiation, conduction, and convection), thermal load, and heat conservation (insulation and reduced openings that reduce heat loss). We present results on temperatures recorded in the newly designed model, the Ncube PH1 tested in 2019 (n_classic_ = 11, n_ncube PH1_ = 8), comparing them to temperatures recorded in the most commonly used model, the Classic 4-chamber. Results from the 10 other models are presented in the Supplementary Information only.

For all the experiments, the area around bat box sites was free of trees or any object causing shade, although roof fringes may have caused shade in the second experiment for the southern orientation. We installed bat boxes at the same heights (3–4 m high). We used external temperatures (T_ext_) from either an in situ meteorological station (https://www.davisinstruments.com/product/vantage-vue-wireless-weather-station/) or the nearest Environment Canada station (maximum distance = 1 km; Supplementary Table S8 for the name and location of the station used). As bats tend to roost at the top of bat boxes, iButtons were placed in the top quarter of each bat box (one in the third chambers from the front in the Classic and two in the Ncube PH1 in the main and lower chambers). The vertical temperature gradient in bat boxes was only verified in the Rocket PH1, the longest model, expected to be more susceptible to vertical temperature gradients. Temperature varied (up to 7.5 °C) between the upper and lower part of the rocket box, but this variance decreased as the internal temperature increased. At high temperatures, this variance was often near zero and highest vertical temperature differences occurred mostly during the night, between 2000 and 0000 (Supplementary Fig. S7). We also recorded temperature variation among the four chambers of the Classic model in 2017 at two sites on poles. The average hourly temperature in the four chambers did not vary during the night but varied during the day, with the second and third chambers being colder than the first and fourth chambers (Supplementary Fig. S8).

Bat boxes were monitored at least every month to verify the absence of bats in boxes (monitoring; guano, bat sighting or hearing) to ensure a colony did not occupy bat box permanently, which would have affected the internal temperatures of bat boxes. During the four years of monitoring, no colony inhabited the bat boxes and we only detected two individuals in two different bat boxes. The occasional presence of individual bats would not change the overall internal temperature profiles of the bat boxes as those individuals were likely males or non-reproductive females that often use torpor to save energy^83–85^. Furthermore, we carefully investigated the data for signatures of anomalous increases in temperature indicative of bat presence which we did not find.

### Statistical analysis

To compare non-linear daily temperature profiles and evaluate differences among treatments of each experiment (orientations, mountings, and designs), we used general additive mixed models (GAMMs). We modeled internal temperature differences among orientations for Classic models by including bat box T_int_ facing south, south-east, east, and west as response variables, orientation, time, date, T_ext_, and structure as fixed effects, and location and bat box identity as random factors. We modeled internal temperature differences among structures for Classic models by including bat box T_int_ mounted on poles, heated, and non-heated buildings as response variables, structure, time, date, T_ext,_ and orientation as fixed effects, and location and bat box identity as random factors. We evaluated differences in internal temperature between the Classic and the Ncube PH1 models by including bat box T_int_ of the Classic and the Ncube PH1 as a response variable, time, model, week, T_ext_, and structure as fixed effects, and location and bat box identity as random factors (see Supplementary Table S9 for statistical model descriptions).

Because of the high daily temperature variance among sites and years in the design dataset, we used mean hourly temperatures over 14 days instead of every day to reduce confidence intervals, which helped to detect differences among bat box models. We equally divided time between day (700–1800) and night (1800–700), which allows a finer investigation of bat box thermal patterns with and without sun exposure (presumably warmer during the day and colder during the night). This division also roughly corresponded to periods when females are more likely to be in the roost (day) and partly away (night). We used mcgv package in R for all analysis and used a significance threshold of ≤ 0.05 for all tests. To show the temperature variation among bat boxes, we also provided the percentage of time below, between and above EOTR, and minimal and maximal internal temperatures (T_int-min_, and T_int-max_) for each experiment.

### Bioenergetic modeling

Reproductive female little brown bats select roosts that reduce energy expenditure, increase time spent in normothermia and facilitate torpor in the early morning^73,86^. We used bioenergetic models to predict energy expenditure of the little brown bat in Classic and Ncube PH1 models, based on their respective T_int_ and T_ext_ recorded at cooler, intermediate, and warmer sites. As shown in other studies^32,42^, we assumed that once temperatures reached 40 °C or higher, bats would exhibit behavioral thermoregulation and systematically go down to select a cooler space to avoid detrimental effects of overheating. Therefore, we selected the T_int_ of the Ncube PH1 main chamber when equal or lower than 40 °C and the lower chamber when above 40 °C. T_int_ of the Classic was based on the middle chamber (chamber 3 from the front) at all times since external chambers acted as insulation, reducing overheating during the day while similar to the other chamber temperatures during at night (Supplementary Fig. S8).

We estimated daily energy expenditure during gestation and lactation periods based on the reproductive phenology, activity budgets, foraging flight costs, mean torpor duration per day and typical diet of the little brown bat in Quebec^20,32,74,77^. We calculated daily energy expenditure (Table 3; converted into kJ h^−1^) from the sum of (1) normothermic energy expenditure (E_norm_), (2) energy expenditure during torpor (E_tor_) including cooling and torpor phases, and (3) energy costs of active arousals from torpor (E_ar_). We then added the energy expenditure for foraging flights (in kJ h^−1^) to the estimates. After visually assessing for data normality, we used a Welch’s paired t-test in R to determine if there was a significant difference in daily energy expenditure between the Classic and the Ncube PH1 2019 during gestation and lactation at cooler, intermediate, and warmer sites using a significance threshold of ≤ 0.05.

**Table 3:** Bioenergetic modeling to estimate the theoretical energy expenditure of reproductive female bats using the Classic and Ncube PH1 bat boxes.

Parameters used in the bioenergetic models to quantify energy expenditure of little brown bats in Classic and newly designed Ncube PH1 bat boxes		
Energy parameters	Value	Reference
Mass (Mb)	8.47 g	^87^
Basal metabolic rate (BMR)	1.44 ml O2 g^−1^ h^−1^	^88^
Minimal torpid metabolic rate (TMR_min_)	0.03 ml O2 g^−1^ h^−1^	^20,89–92^
Normothermic temperature (T_norm_)	35 °C	^20,90–92^
Lower critical temperature (T_lc_)	32 °C	^20,90,91,93^
Upper critical temperature (T_uc_)	37 °C	^20,28^
Minimal torpid temperature (T_tor-min_)	2 °C	^20,85–87^
Normothermic conductance below the lower critical temperature (C_lnorm_)	0.2638 ml O2 g^−1^ °C^−1^	^20,86,87,89^
Normothermic conductance above the upper critical temperature (C_unorm_)	0.4978 ml O2 g^−1^ °C^−1^	^85^
Torpid conductance (C_tor_) or (C_lct_)	0.055 ml O2 g^−1^ °C^−1^	^20,86–88^
Change in torpid metabolic rate (TMR) over a 10 °C change in Ta (Q_10_)	1.6 + 0.26T_a_ − 0.006 T_a_^2^	^20,89–91^
Specific heat capacity of tissue (S)	0.131 ml O2 g^−1^ °C^−1^	^20,90–92^

Little brown bat reproductive parameters	Value	Reference
Gestating mean torpor duration per day	133 min	^20,73^
Lactating mean torpor duration per day	334 min	^20,73^
Gestation period	May 15–June 30. We used a 2 weeks delay for cooler sites	^32^
Lactation period	June 16–August 1. We used a 2 weeks delay for cooler sites	^32^
Gestation activity budget	Foraging: 2 bouts of 20 min, resting in the roost: 1080 min, resting outside: 120 min	^32^
Lactation activity budget	Foraging: 3 bouts of 105 min, resting in the roost: 2 bouts of 60 min and 1 bouts of 1080 min, resting outside: 0 min	^32^
Foraging flight during gestation by 8.47 g little brown bat	4.20 kJ h^−1^	^77^
Foraging flight during lactation by 8.47 g little brown bat	3.90 kJ h^−1^	^77^
Typical diet for little brown bat	71.2% protein, 18.4% fat and 8.8% carbohydrate	^77^

**Bioenergetic formulas used to quantify energy expenditure of little brown bats**		
**(1) Calculating normothermic energy expenditure (E**_***norm***_**)**		
The normothermic energy expenditure varies with ambient temperature, *T*_*a*_, according to a metabolic response curve using the following equations^20,88–90^		
when T_a_ > T_lc_; E_norm_ = BMR + (T_lc_ − T_a_)*C_lnorm_		
when T_a_ < T_uc_; E_norm_ = BMR + (T_a_ − _uc_)*C_unorm_		
when T_a_ ≥ T_lc_ ≤ T_uc_; E_norm_ = BMR		
where *BMR* is the basic metabolic rate, *T*_*lc*_ is the lower critical temperature, *T*_*uc*_ is the upper critical temperature, *C*_*lnorm*_ is the thermal conductance in normothermia below the lower critical temperature, and *C*_*unorm*_ is the thermal conductance in normothermia above the upper critical temperature		
**(2) Quantify predicted energy expenditure during torpor depending on whether T**_***a***_** was lower or higher than T**_***tor-min***_		
During torpor, metabolic rate, *TMR*, and body temperature decline with *T*_*a*_ until a lower ambient set-point temperature, *T*_*tor-min*_, is reached, after which torpor body temperature is defended (that is, remains constant) and consequently *TMR* increases. Thus, *TMR* varies with temperature according to^20,90–92^		
when T_a_ > T_tor-min_; E_tor_ = TMR_min_*Q_10_^((T_a_ − T_tor-min_)/10)		
when T_a_ ≤ T_tor-min_; E_tor_ = TMR_min_ + (T_tor-min_ − T_a_)*C_t_		
where *Q*_*10*_ represents the change in torpor metabolism resulting from a 10 °C change in *T*_*a*_, and *C*_*t*_ represents torpor conductance below *T*_*tor-min*_		
Cooling phase = 67.2% of active arousal		
**(3) Calculating predicted energetic cost for active arousal**		
The energetic cost of arousals *E*_*ar*_ is a simple function of the required increase in body temperature from *T*_*tor*_ to normothermic levels, *T*_*norm*_, and the specific heat capacity *S* of the bat’s tissues^89–91^		
E_ar_ = (T_norm_ -T_tor_)*S		
**(4) Conversion from mass-specific Vo2 into SI (energy expenditure)**^20,94^		
Heat Production = (17.71P + 20.93C + 19.55L)*mass-specificVo_2_		

## Supplementary Information

Supplementary Information.

## Data Availability

The datasets generated during and/or analysed during the current study are available in the Open Science Framework repository, https://osf.io/kh2zw/?view_only=0656ff50af394a81a842f0c42a2c2b44.
