# Supplementary material for: Using mounting, orientation, and design to improve bat box thermodynamics in a northern temperate environment
Source: Sci Rep. 2021 Apr 8;11:7728. doi: 10.1038/s41598-021-87327-3 (PMC8032723; doi:10.1038/s41598-021-87327-3)
Supplement: Supplementary file 1 — Supplementary Information. [file 41598_2021_87327_MOESM1_ESM.pdf]

Supplementary Information. Using thermodynamics to improve bat boxes in northern temperate environment. Amélie Fontaine, Anouk Simard, Bryan Dubois, Julien Dutel, Kyle H. Elliott.

Table S1. Description of bat box models tested in Quebec, Canada, from 2016 to 2019. Model names refer to their shape and number of passive heating (PH) zones (1, 2 or 3 reflexives faces). An iButton was placed in the top quarter of each bat box except for the newly designed models where one iButton was placed in the main or warm chamber and a second one in the lower or cold chamber.

**Model**		**Image(s)**	**Dimensions and materials**	**Description**	**Years and number of bat boxes tested on poles and buildings**
**Traditional**					
1	Classic (4-chambers)	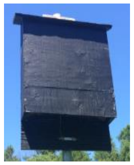 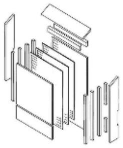	78x43x11 cm<br>wood	Rectangular shape. Commonly used in North America. Developed by Bat Conservation International. Cheap and easy to build.	2016 (n_{pole}=2, n_{build}=3)<br>2017 (n_{pole}=10, n_{build}=16)<br>2018 (n_{pole}=6, n_{build}=5)<br>2019 (n_{pole}=6, n_{build}=5)
2	European (1-chamber)	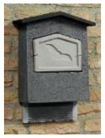	44x29x9 cm<br>woodcement	Rectangular shape. Chillon bat box from wildcare. Woodcement commonly used in Europe. This material has similar properties than wood, but more durable and heavier.	2016 (n_{pole}=2, n_{build}=2)<br>2017 (n_{pole}=2, n_{build}=2)<br>2018 (n_{pole}=2, n_{build}=2)<br>2019 (n_{pole}=2, n_{build}=2)
**Rocket**					
3	Insulated rocket (1-chamber)	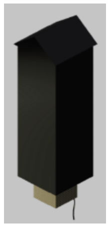	120x34x34 cm<br>wood and 2.5 cm thick styrofoam insulation	The rocket model has an elongated shaped that allow a vertical gradient of temperature. One big chamber evolving around a closed space in the middle used to insert a pole or a heat mat.	2016 (n_{pole}=1, n_{build}=1)<br>2017 (n_{build}= 1)<br>2018 (n_{pole}=1)<br>2019 (n_{build}=1)
4	Insulated heated rocket (1-chamber)	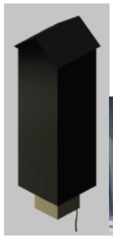 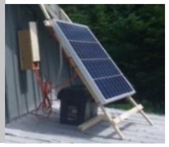	120x34x34 cm<br>wood, 2.5 cm thick styrofoam insulation, heated mat, and a solar kit (undulator, timer, converter, battery, and solar panel)	The rocket model has an elongated shaped that allow a vertical gradient of temperature. One big chamber evolving around a closed space in the middle used to insert a pole or a heat mat. Developed by Genivar in 2011 and adapted to solar energy by Quebec MFFP in 2016. Considering the capacity limit of the battery, a timer was set to turn on the heated mat for 30 minutes three times per night: 12:00AM, 2:00AM, and 4:00AM.	2016 (n_{pole}=1, n_{build}=1)<br>2017 (n_{pole}=2, n_{build}=1)<br>2018 (n_{pole}=1, n_{build}=2)<br>2019 (n_{pole}=2, n_{build}=1)

[1] *Email:* amelie.fontaine@mail.mcgill.ca
[2] *Current affiliation: Natural Resource Sciences, McGill University, 21,111 Lakeshore road, St-Anne-de-Bellevue, Qc, Can.*

**Newly designed model**					
5	Biclimate.0 PH1 (2-chambers)	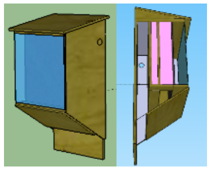	60x30x20 cm wood, 2.5 cm thick styrofoam insulation, permabase cement board, and plexiglass	One "cold" chamber at the back. In the middle, the insulated "hot" chamber stands against a frontal passive heating zone that conducts the heat inside. The passive heating zone is made of plexiglass, an empty space (air) and a conductive material (permabase cement board).	2016 (n_{pole}=2, n_{build}=2)<br>2017 (n_{pole}=2, n_{build}=2)<br>2018 (n=0)<br>2019 (n=0)
6	Biclimate PH1 (2-chambers)	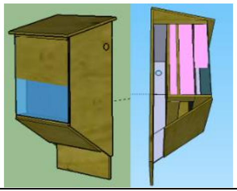	81x43x23 cm wood, 2.5 cm thick styrofoam insulation, permabase cement board, and plexiglass	Same as the Biclimate model but bigger dimensions and a reduction of the passive heating zone by two thirds. at Transition Énergétique Quebec.	2017 (n_{pole}=3, n_{build}=4)<br>2018 (n_{pole}=3, n_{build}=4)<br>2019 (n=0)
7	Rocket PH2 (1-chamber)	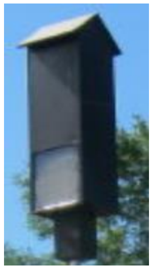	120x34x34 cm wood, 2.5 cm thick styrofoam insulation, plexiglass, and permabase cement board	Same as the insulated rocket model, with the addition of two passive heating zones of 25cmx25cm at the bottom facing east and west. Designed for pole.	2017 (n_{pole}=4)<br>2018 (n_{pole}=4)<br>2019 (n_{pole}=4)
8	Classic PH2 (3-chambers)	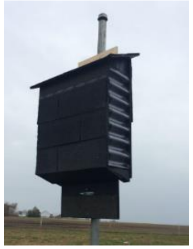	65x58x20 cm wood, 2.5 cm thick styrofoam insulation, clapboards, corrugated PVC panel, and permabase cement board,	Same shape as the classic model but insulated at front and the back and covered with clapboards. Reduction of the entrance to one inch wide. Two passive heating zones on each side, facing east and west, made of corrugated plexiglass to increase the contact surface, an empty space (air) and a conductive material (permabase cement board). Designed for pole.	2018 (n_{pole}=3)<br>2019 (n_{pole}=3)

[1] *Email:* amelie.fontaine@mail.mcgill.ca
[2] *Current affiliation: Natural Resource Sciences, McGill University, 21,111 Lakeshore road, St-Anne-de-Bellevue, Qc, Can.*

9	Classic PH1 (3-chambers)	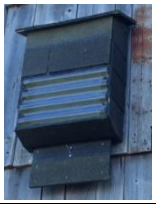	65x53x24 cm wood, 2.5 cm thick styrofoam insulation, plexiglass, and permabase cement board	Same as the Classic PH2 2018 but with only one passive heating zone at the front facing east. Designed for building.	2018 (n_{build}=4) 2019 (n_{build}=4)
10	Ncube.0 PH1 (3-chambers)	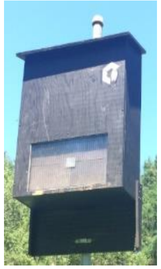 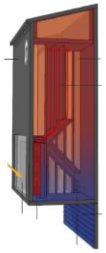	77x52x24 cm wood, 2.5 cm thick styrofoam insulation, plexiglass, and clay brick	Similar shape as the classic PH1 model, but fully insulated. Use of a reduced chicane entrance (2.5 x 10.2 cm). One passive heating zone at the front facing east, overlapping with the entrance preferably facing east. The passive heating zone is made of a high-core corrugated plexiglass, an empty space (air) and a conductive material (clay brick) slowly diffuse the heat inside the three chambers. Convection principle due to an opening at the top of zone. Removable insulated roof. Designed for building.	2018 (n_{pole}=2, n_{build}=5) 2019 (n_{pole}=1, n_{build}=5)
11	Ncube.0 PH3 2018 (4-chambers)	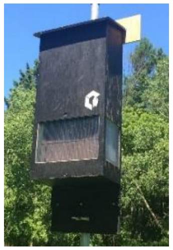 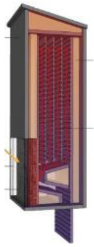	92x41x24 cm wood, 2.5 cm thick styrofoam insulation, plexiglass, and clay brick, light cement panel	Same as the Ncube PH1 2018, but with three passive heating zones facing east, south, and west overlapping with the entrance and the chambers. Conduction principle due to the fixation of the brick on a light cement panel. Removable insulated roof. Designed for pole.	2018 (n_{pole}=2, n_{build}=5) 2019 (n_{build}=5)
12	Ncube PH1 (3-chambers)	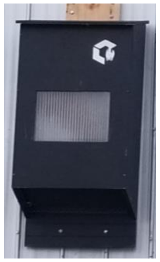 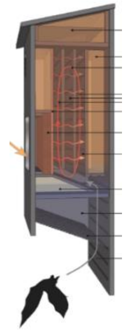	92x41x24 cm wood, 5 cm thick styrofoam insulation, plexiglass, and clay brick, light cement panel	Improved version Ncube PH1 2018 and Ncube PH3 2018 with thicker insulation (10.2 vs. 5 cm). Removable insulated roof. A cool zone at the bottom of the entrance had been added made of 3 wood wall and a fence where bats could safely go if overheating in the main chambers. One passive heating zone at the front facing east overlapping with the main chambers only. Heating zone using conduction principle.	2019 (n_{pole}=4, n_{build}=4)

[1] *Email:* amelie.fontaine@mail.mcgill.ca
[2] *Current affiliation: Natural Resource Sciences, McGill University, 21,111 Lakeshore road, St-Anne-de-Bellevue, Qc, Can.*

Table S2. Parameter estimates, standard error (SE), *t* value, and the probability of observing any value equal or larger than *t* from generalized additive mixed model accounting for time, date, external temperature, orientation, site, and individual bat box identity for T_{int} for the mounting experiment.

	**Estimate**	**SE**	***t* value**	**Pr(>|*t*|)**
Intercept	18.110	2.039	8.882	< 2e-16 ***
Heated building	3.176	0.360	8.815	< 2e-16 ***
Non heated building	3.556	0.360	9.871	< 2e-16 ***
Pole	3.570	0.360	9.911	< 2e-16 ***

Table S3. Adjusted p-values of the mounting comparisons from generalized additive mixed model accounting for time, date, external temperature, orientation, site, and individuals bat box identity. Asterisks (*) represent significative T_{int} differences among mountings during the day and night. P-values have been adjusted for multiple comparisons using the R function wald_gam.

**Mounting comparison**	**Heated building**		**Non-heated building**		**Pole**	
	**Day**	**Night**	**Day**	**Night**	**Day**	**Night**
Heated building			0.326	0.740	0.050*	0.009**
Non-heated building	0.326	0.740			0.326	0.023*
Pole	0.050*	0.009**	0.326	0.023*		

[1] *Email:* amelie.fontaine@mail.mcgill.ca
[2] *Current affiliation: Natural Resource Sciences, McGill University, 21,111 Lakeshore road, St-Anne-de-Bellevue, Qc, Can.*

Table S4. Parameter estimates, standard error (SE), *t* value, and the probability of observing any value equal or larger than *t* from generalized additive mixed model accounting for time, date, external temperature, mounting, site, and individual bat box identity for T_{int} for the orientation experiment.

	**Estimate**	**SE**	***t* value**	**Pr(>|*t*|)**
Intercept	-2.041e-05	3.840e-04	-0.053	0.958
External	-3.618	0.597	-6.057	1.4e-09 ***
South	-1.178	0.598	-1.972	0.049 *
South-East	0.214	0.299	0.716	0.474
West	-0.145	0.597	-0.243	0.808
Non heated building	0.401	0.597	0.671	0.502
Pole	0.141	0.598	0.235	0.814
Year	0.011	4.454e-04	24.253	< 2e-16 ***
External:Non heated building	-0.298	0.810	-0.368	0.713
South:Non heated building	0.448	0.845	0.530	0.596
West:Non heated building	-0.509	0.845	-0.603	0.547
External:Pole	-0.037	0.81	-0.046	0.963
South:Pole	0.670	0.845	0.792	0.428
South-East:Pole	0.214	0.299	0.716	0.474

Table S5. Adjusted p-values of the orientation comparisons from generalized additive mixed model accounting for time, date, external temperature, mounting, site, and individual bat box identity. Asterisks (*) represent significative T_{int} differences between orientations during the day and night. P-values have been adjusted for multiple comparisons using the R function wald_gam.

**Orientation comparison**	**Heated building**		**Non-heated building**		**Pole**	
	**Day**	**Night**	**Day**	**Night**	**Day**	**Night**
East-South	0.046 *	0.274	0.023*	0.876	0.023 *	0.642
East-West	0.033*	0.007 **	0.014*	0.417		
South-West	0.310	0.106	0.787	0.334		
East-SouthEast					0.232	0.977
South-SouthEast					0.283	0.624

[1] *Email:* amelie.fontaine@mail.mcgill.ca
[2] *Current affiliation: Natural Resource Sciences, McGill University, 21,111 Lakeshore road, St-Anne-de-Bellevue, Qc, Can.*

Table S6. Parameter estimates, standard error (SE), *t* value, and the probability of observing any value equal or larger than *t* from generalized additive mixed model accounting for time, week, year, mounting, external temperature, site, and individual bat box identity for T_{int} for the design experiment.

	**Estimate**	**SE**	***t* value**	**Pr(>|*t*|)**
Intercept	612.775	275.523	2.224	0.026 *
Biclimate PH1 hot chamber	2.098	0.605	3.467	5.28 e-04 ***
Biclimate PH1 cold chamber	0.080	1.906	0.042	0.967
Biclimate PH1.0 cold chamber	1.110	0.740	1.500	0.134
Biclimate PH1.0 hot chamber	2.994	0.739	4.053	5.06e-05 ***
Classic	1.080	0.508	2.110	0.035 *
Classic PH1	2.152	0.734	2.933	0.003 **
Classic PH2	3.042	0.674	4.514	6.38e-06 ***
European	0.246	0.613	0.401	0.688
External	-2.662	0.497	-5.355	8.60e-08 ***
Ncube PH1.0	2.742	0.707	3.879	1.28 e-04 ***
Ncube PH3.0	3.995	0.703	5.686	1.31e-08 ***
Ncube PH1 lower chamber	-0.270	0.747	-0.362	0.718
Ncube PH1 main chamber	3.968	0.746	5.320	1.05e-07 ***
Heated Insulated Rocket	4.540	1.012	4.485	7.32e-06 ***
Insulated Rocket	-0.293	0.695	-0.421	0.673
Rocket PH2	1.693	0.597	2.834	0.005 **
August 2	-0.485	0.064	-7.613	2.75e-14 ***
July 1	0.195	0.065	3.026	0.002 **
July 2	0.166	0.064	2.586	0.010 **
June 1	-0.171	0.076	-2.260	0.024 *
June 2	0.0149	0.067	0.223	0.823
May 2	-0.798	0.089	-8.925	< 2e-16 ***
September 1	-0.808	0.073	-11.069	< 2e-16 ***
Pole	-0.325	0.236	-1.375	0.169
Year	-0.293	0.137	-2.148	0.032 *

[1] *Email:* amelie.fontaine@mail.mcgill.ca
[2] *Current affiliation: Natural Resource Sciences, McGill University, 21,111 Lakeshore road, St-Anne-de-Bellevue, Qc, Can.*

Table S7. Adjusted p-values of the bat box design comparisons from the generalized additive mixed model accounting for time, week, year, mounting, external temperature, site, and individual bat box identity. Asterisks (*) represent significative T_{int} differences between models during the day and night. P-values have been adjusted for multiple comparisons using the R function wald_gam.

Model comparison	Classic		European		Bilimate 2017 hot chamber		Biclimate 2017 cold chamber		Classic PH1 2018		Classic PH2 2018		Ncube PH1 2018	
	Day	Night	Day	Night	Day	Night	Day	Night	Day	Night	Day	Night	Day	Night
Classic			0,68	0,522	0.006 **	0,909	0,288	0,328	0,403	0.039 *	0,808	0.013 *	0,959	0.013 *
European	0,68	0,522			0.009 **	0,659	0,592	0,782	0,649	0.022 *	0,614	0.010 *	0,732	0.008 **
European V2	0,303	0.077 .	0,243	0,153	0,872	0.093 .	0,144	0,191	0,178	0.008 **	0,406	0.005 **	0,363	0.005 **
Biclimate PH1.0 hot chamber	2.93e-05 ***	0,642	4.54e-05 ***	0,972	0.061 .	0,741	9.28e-06 ***	0,792	1.13e-04 ***	0.048 *	0.002 **	0.028 *	0.001 **	0.025 *
Biclimate PH1.0 cold chamber	0,444	0,698	0,331	0,917	0,278	0,792	0,168	0,74	0,224	0.055 .	0,649	0.033 *	0,566	0.029 *
Biclimate PH1 hot chamber	0.006 **	0,909	0.009 **	0,659			0.001 **	0,463	0.009 **	0.057 .	0.074 .	0.027 *	0.056 .	0.024 *
Biclimate PH1 cold chamber	0,288	0,328	0,592	0,782	0.001 **	0,463			0,993	0.012 *	0,324	0.004 **	0,42	0.004 **
Classic PH1	0,403	0.039 *	0,649	0.022 *	0.009 **	0.057 .	0,993	0.012 *			0,383	0,856	0,467	0,807
Classic PH2	0,808	0.013 *	0,614	0.010 *	0.074 .	0.027 *	0,324	0.004 **	0,383	0,856			0,882	0,946
Ncube PH1.0	0,959	0.013 *	0,732	0.008 **	0.056 .	0.024 *	0,42	0.004 **	0,467	0,807	0,363	0.005 **		
Ncube PH3.0	0,432	6.95e-04 ***	0,338	8.07e-04 ***	0,211	0.002 **	0,15	2.51e-04 ***	0,198	0,339	0,582	0.001 **	0,554	0,457
Ncube PH1 main chamber	0.022 *	0.018 *	0.021 *	0.011 *	0,869	0.030 *	0.006 **	0.005 **	0.013 *	0,82	0,809	0.005 **	0.063 .	0,992
Ncube lower chamber	0,251	0,344	0,466	0,701	0.004 **	0,449	0,771	0,88	0,799	0.019 *	0,133	0,259	0,321	0.007 **
Heated Insulated Rocket	0,951	4.79e-04 ***	0,877	2.97e-04 ***	0,147	8.09e-04 ***	0,628	1.63e-04 ***	0,645	0.056 .	0,388	2.11e-04 ***	0,932	0.079 .
Insulated Rocket	0.006 **	0.086 .	0.034 *	0.045 *	1.27e-05 ***	0,111	0,102	0.027 *	0,161	0,742	0.023 *	0.014 *	0.030 *	0,558
Rocket PH2	0,1	0.063 .	0,324	0.043 *	2.14e-04 ***	0.097 .	0,631	0.017 *	0,705	0,587	0.089 .	0.012 *	0,224	0,397
External	1.07e-13 ***	1.83e-04 ***	4.70e-07 ***	0.036 *	2.16e-16 ***	0.009 **	1.24e-05 ***	0.080 .	7.19e-04 ***	2.09e-05 ***	6.90e-04 ***	0,56	7.68e-06 ***	1.69e-06 ***

[1] *Email:* amelie.fontaine@mail.mcgill.ca
[2] *Current affiliation: Natural Resource Sciences, McGill University, 21,111 Lakeshore road, St-Anne-de-Bellevue, Qc, Can.*

Model comparison (Continued)	Ncube PH3 2018		Ncube 2019 main chamber		Ncube 2019 lower chamber		Heated Rocket		Non Heated Rocket		Rocket PH2 2017		External	
	Day	Night	Day	Night	Day	Night	Day	Night	Day	Night	Day	Night	Day	Night
Classic	0,432	6.95e-04 ***	0.022 *	0.018 *	0,251	0,344	0,951	4.79e-04 ***	0.006 **	0.086 .	0,1	0.063 .	1.07e-13 ***	1.83e-04 ***
European	0,338	8.07e-04 ***	0.021 *	0.011 *	0,466	0,701	0,877	2.97e-04 ***	0.034 *	0.045 *	0,324	0.043 *	4.70e-07 ***	0.036 *
European V2	0,582	0.001 **	0,809	0.005 **	0,133	0,259	0,388	2.11e-04 ***	0.023 *	0.014 *	0.089 .	0.012 *	6.90e-04 ***	0,56
Biclimate PH1.0 hot chamber	0.008 **	0.004 **	0,146	0.028 *	5.91e-05 ***	0,714	0.008 **	7.68e-04 ***	3.05e-08 ***	0.087 .	2.22e-06 ***	0.098 .	< 2e-16 ***	0.093 .
Biclimate PH1.0 cold chamber	0,957	0.004 **	0,285	0.032 *	0,154	0,669	0,588	9.04e-04 ***	0.008 **	0.099 .	0.086 .	0,112	2.36e-06 ***	0.079 .
Biclimate PH1 hot chamber	0,211	0.002 **	0,869	0.030 *	0.004 **	0,449	0,147	8.09e-04 ***	1.27e-05 ***	0,111	2.14e-04 ***	0.097 .	2.16e-16 ***	0.009 **
Biclimate PH1 cold chamber	0,15	2.51e-04 ***	0.006 **	0.005 **	0,771	0,88	0,628	1.63e-04 ***	0,102	0.027 *	0,631	0.017 *	1.24e-05 ***	0.080 .
Classic PH1	0,198	0,339	0.013 *	0,82	0,799	0.019 *	0,645	0.056 .	0,161	0,742	0,705	0,587	7.19e-04 ***	2.09e-05 ***
Classic PH2	0,641	0,396	0.086 .	0,956	0,257	0.008 **	0,845	0.070 .	0.020 *	0,604	0,145	0,415	1.23e-06 ***	1.30e-06 ***
Ncube PH1.0	0,554	0,457	0.063 .	0,992	0,321	0.007 **	0,932	0.079 .	0.030 *	0,558	0,224	0,397	7.68e-06 ***	1.69e-06 ***
Ncube PH3.0			0,207	0,467	0,122	7.79e-04 ***	0,603	0,224	0.007 **	0,197	0.056 .	0.089 .	2.23e-07 ***	1.61e-08 ***
Ncube PH1 main chamber	0,207	0,467			0.005 **	0.009 **	0,145	0.084 .	9.40e-05 ***	0,572	0.002 **	0,421	9.44e-11 ***	4.86e-06 ***
Ncube lower chamber	0,122	7.79e-04 ***	0.005 **	0.009 **			0,516	2.67e-04 ***	0,254	0.037 *	0,924	0.034 *	0.003 **	0,225
Heated Insulated Rocket	0,603	0,224	0,145	0.084 .	0,516	2.67e-04 ***			0,122	0.031 *	0,44	0.017 *	0.004 **	8.62e-07 ***
Insulated Rocket	0.007 **	0,197	9.40e-05 ***	0,572	0,254	0.037 *	0,122	0.031 *			0,228	0,853	0.091 .	7.27e-05 ***
Rocket PH2	0.056 .	0.089 .	0.002 **	0,421	0,924	0.034 *	0,44	0.017 *	0,228	0,853			1.35e-04 ***	3.66e-06 ***
External	2.23e-07 ***	1.61e-08 ***	9.44e-11 ***	4.86e-06 ***	0.003 **	0,225	0.004 **	8.62e-07 ***	0.091 .	7.27e-05 ***	1.35e-04 ***	3.66e-06 ***		

[1] *Email:* amelie.fontaine@mail.mcgill.ca
[2] *Current affiliation: Natural Resource Sciences, McGill University, 21,111 Lakeshore road, St-Anne-de-Bellevue, Qc, Can.*

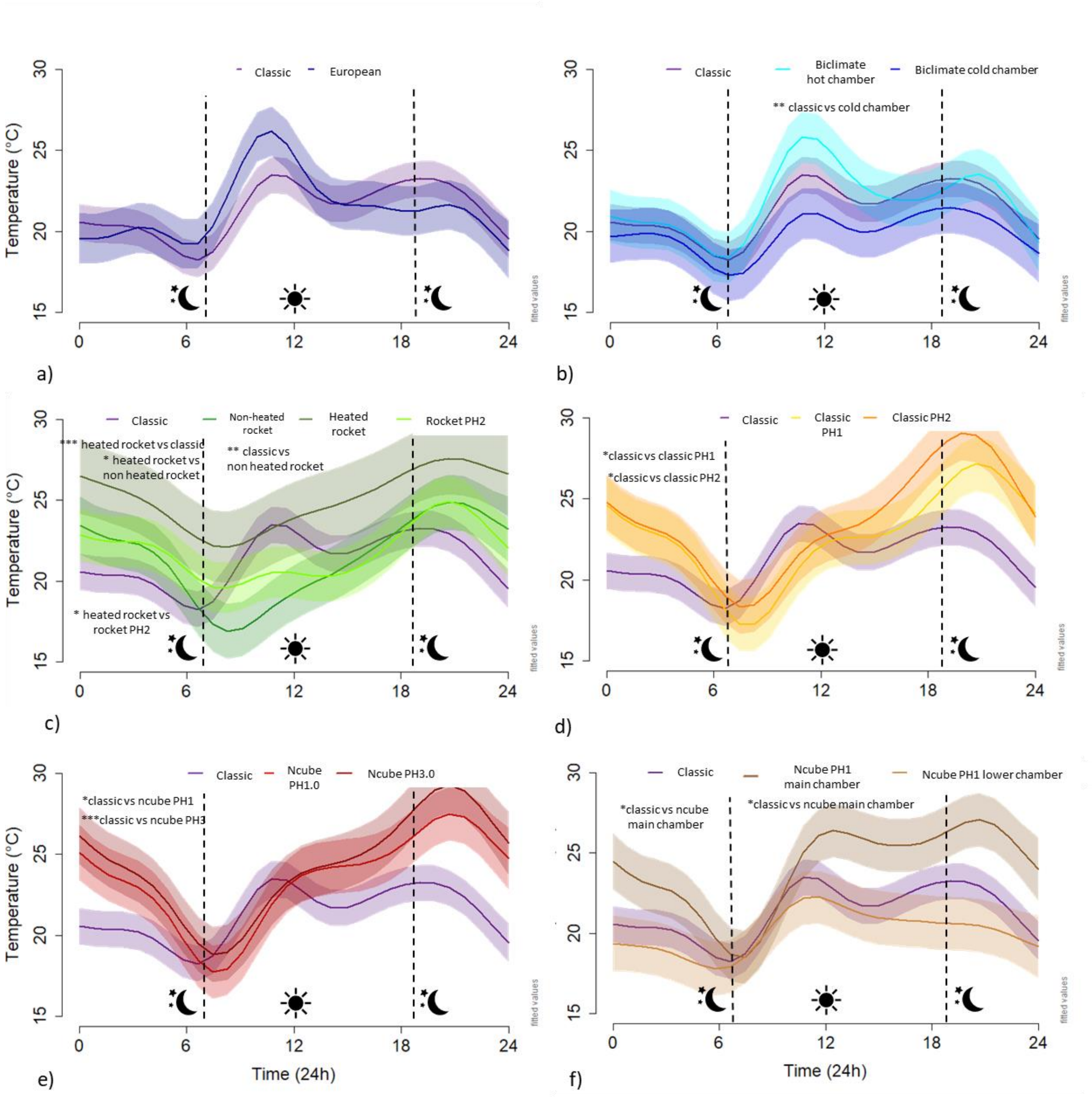

Figure S1. Estimated hourly patterns for different bat box designs compared with the Classic models. The estimated values are based on a generalized additive mixed model accounting for time, week, year, mounting, external temperature, site, and individual bat box identity. Values of fixed factors have been set to: week = first half of July, year = 2019, mounting = building, external temperature = 18°C. a) European, b) Biclimate cold and hot chambers, c) Heated Rocket and Rocket PH2, d) Classic PH1 and PH2, e) Ncube PH1.0 and PH3.0, and f) Ncube PH1 main and lower chambers. The dotted vertical lines represent the separation between the day and the

[1] *Email:* amelie.fontaine@mail.mcgill.ca
[2] *Current affiliation: Natural Resource Sciences, McGill University, 21,111 Lakeshore road, St-Anne-de-Bellevue, Qc, Can.*

night. The asterisks (*) represent significant differences between models during the day and night.

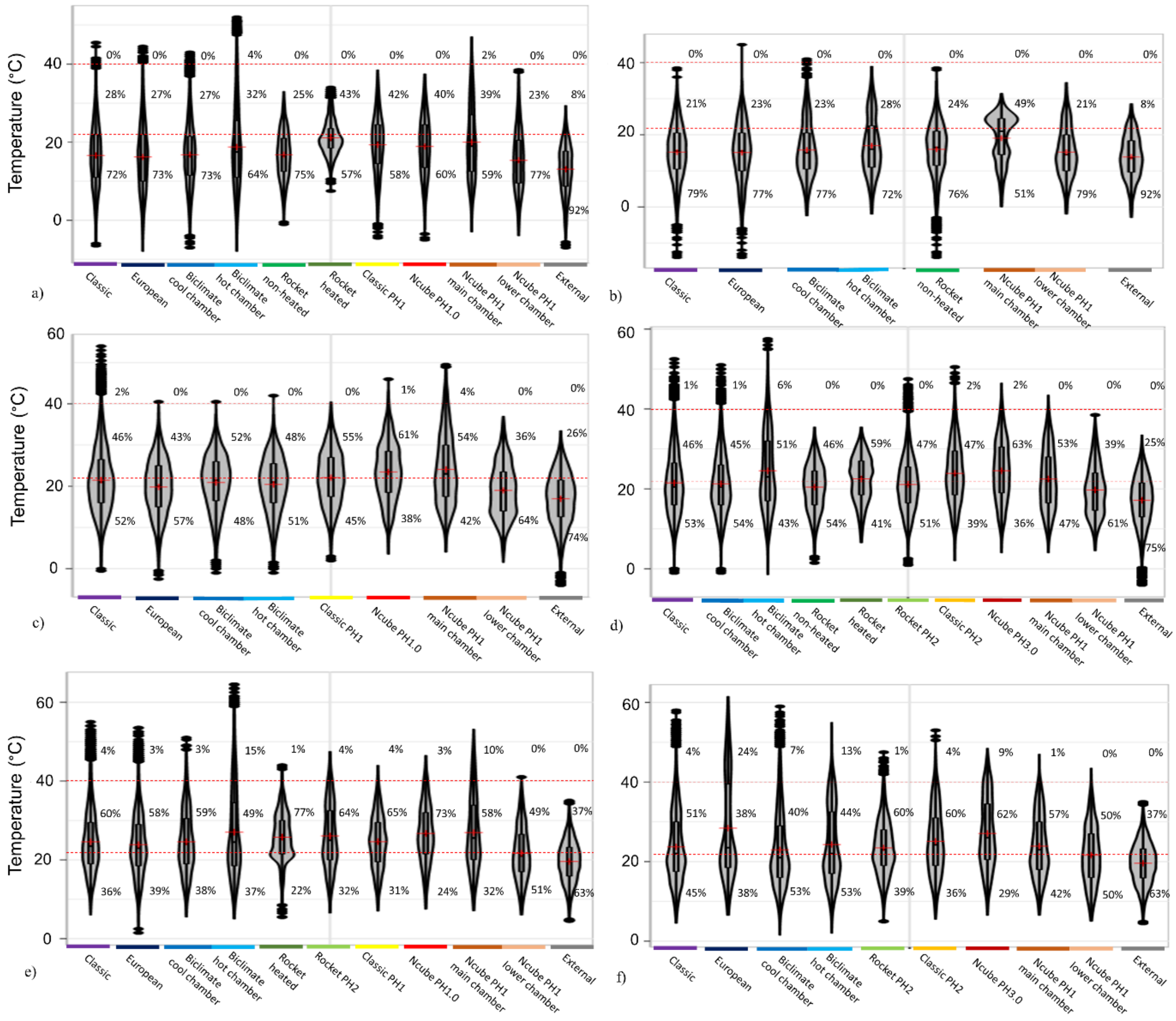

Figure S2. Variation of internal temperatures for all models from mid-May to mid-September 2017-2019 per climate and mounting type in Quebec, Canada: a) cooler sites on buildings, b) cooler sites on poles, c) intermediate sites on buildings, d) intermediate sites on poles, e) warmer sites on buildings, f) warmer sites on poles. The violins represent the temperature range of each model with the minimal and maximal temperature represented by the bottom and the top of the violin respectively. The width of the violin represents the distribution of the data. The black horizontal bar represents the median and the red cross the mean value. The red dotted lines represent the extended optimal temperature range of 22-40 °C. The percentages below, in between, and over the extended optimal temperature range are represented beside each violin.

[1] *Email:* amelie.fontaine@mail.mcgill.ca
[2] *Current affiliation: Natural Resource Sciences, McGill University, 21,111 Lakeshore road, St-Anne-de-Bellevue, Qc, Can.*

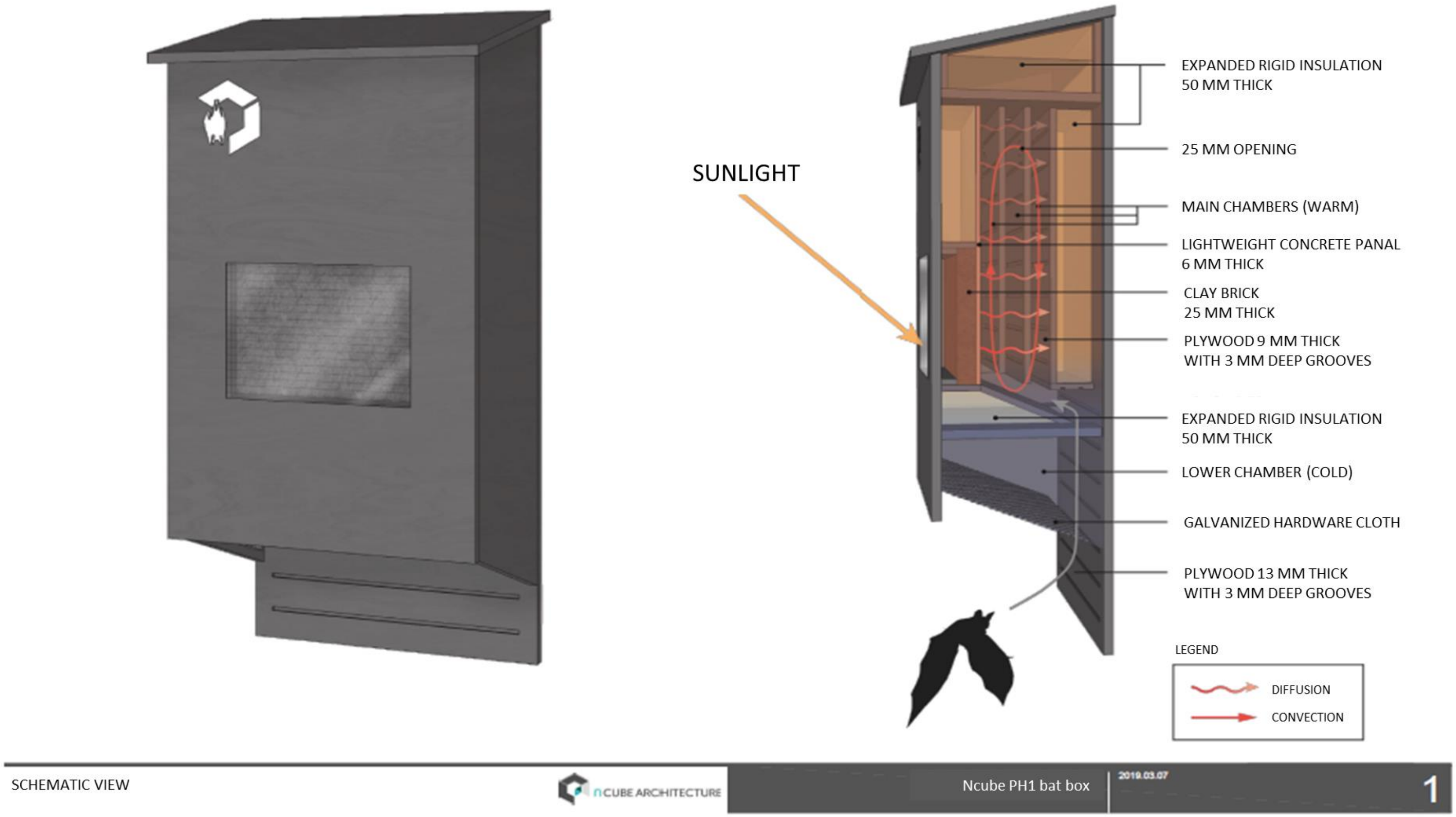

Figure S3. Schematic view of the Ncube PH1 2019. The improved Ncube PH1 2020 plan is available in French and English on Batwatch.ca.

[1] *Email:* amelie.fontaine@mail.mcgill.ca
[2] *Current affiliation: Natural Resource Sciences, McGill University, 21,111 Lakeshore road, St-Anne-de-Bellevue, Qc, Can.*

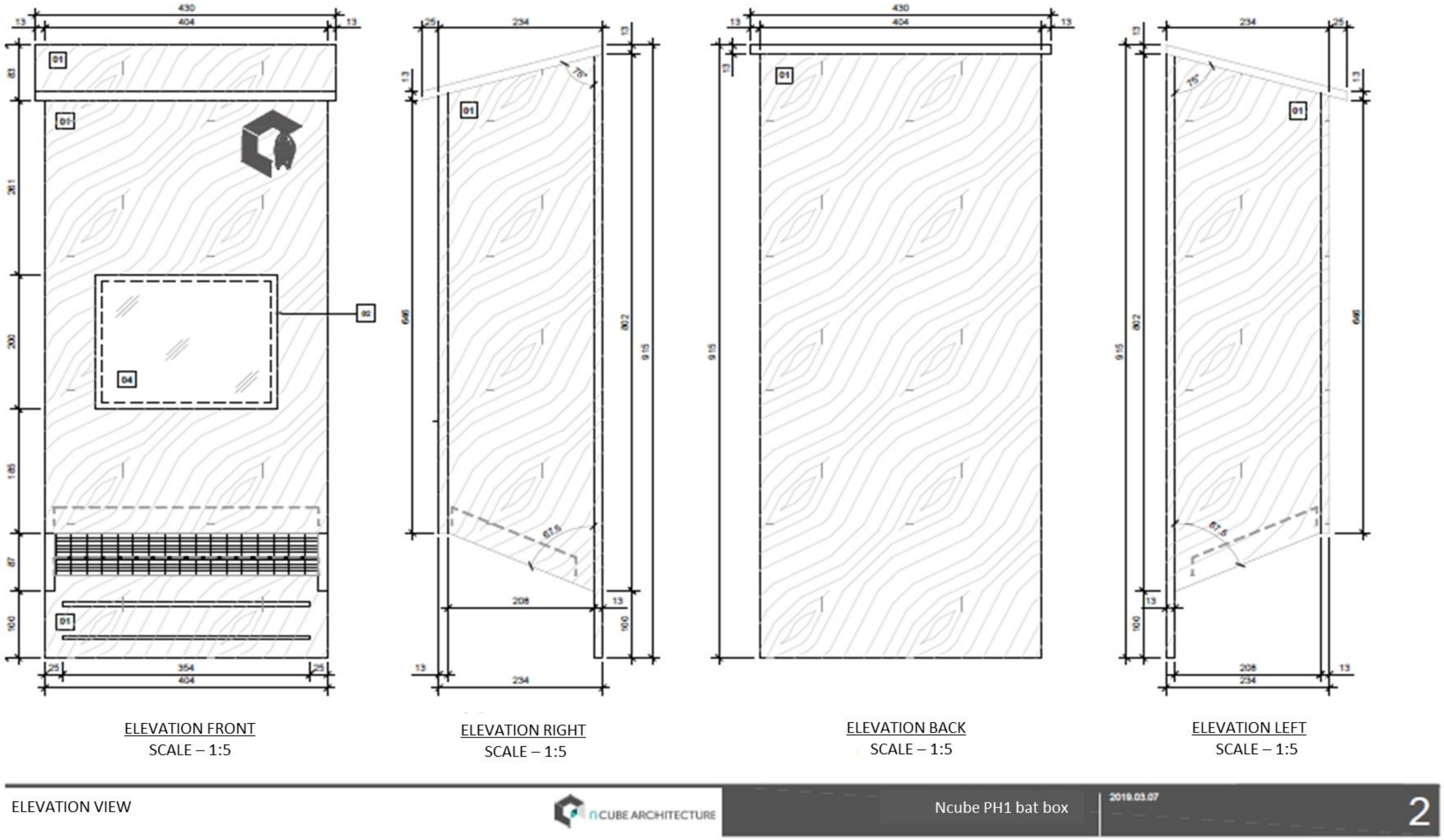

Figure S4. Design plan of the Ncube PH1 2019. Elevation view.

[1] *Email:* amelie.fontaine@mail.mcgill.ca
[2] *Current affiliation: Natural Resource Sciences, McGill University, 21,111 Lakeshore road, St-Anne-de-Bellevue, Qc, Can.*

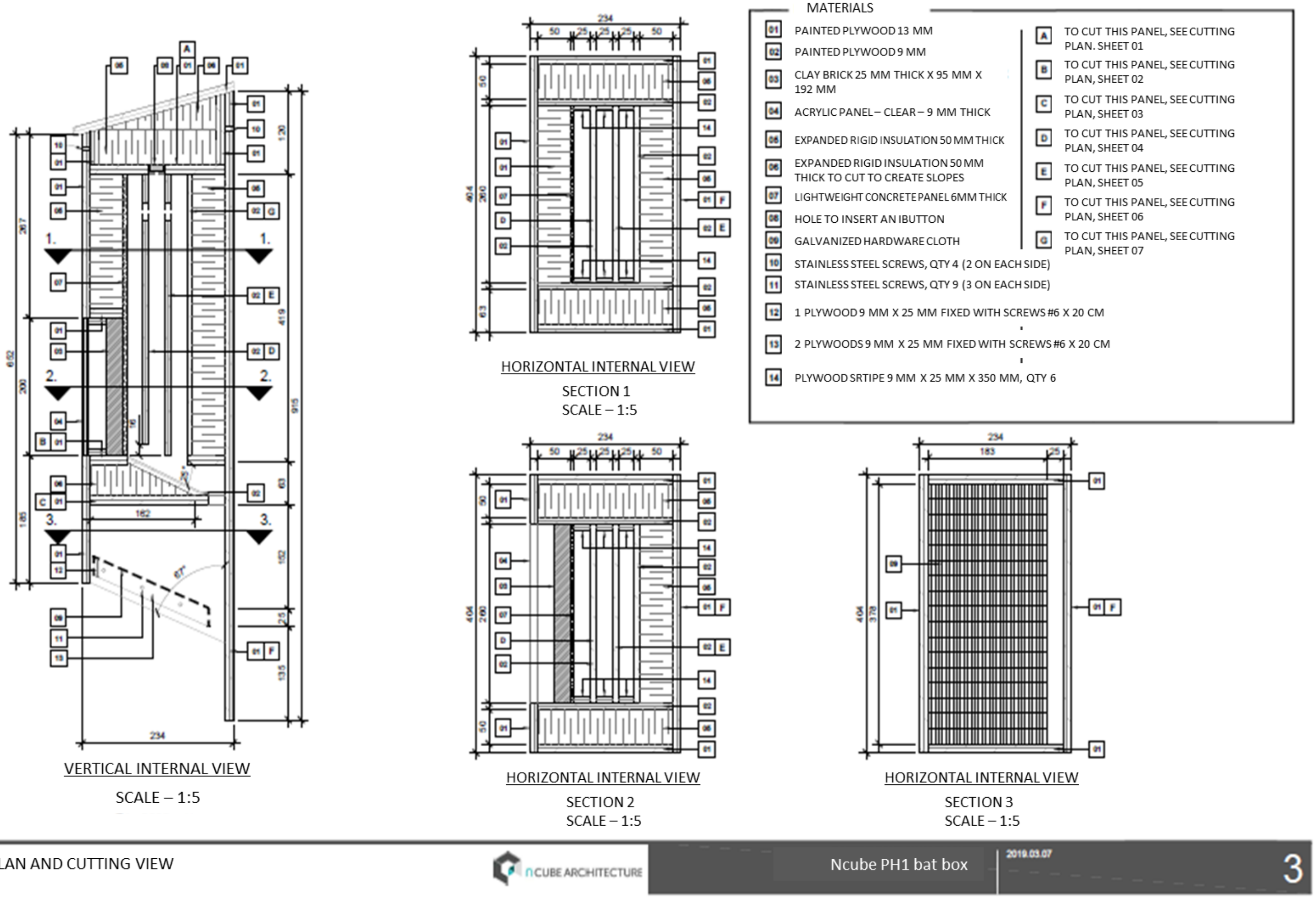

Figure S5. Design plan of the Ncube PH1 2019. Cutting plane view.

[1] *Email:* amelie.fontaine@mail.mcgill.ca
[2] *Current affiliation: Natural Resource Sciences, McGill University, 21,111 Lakeshore road, St-Anne-de-Bellevue, Qc, Can.*

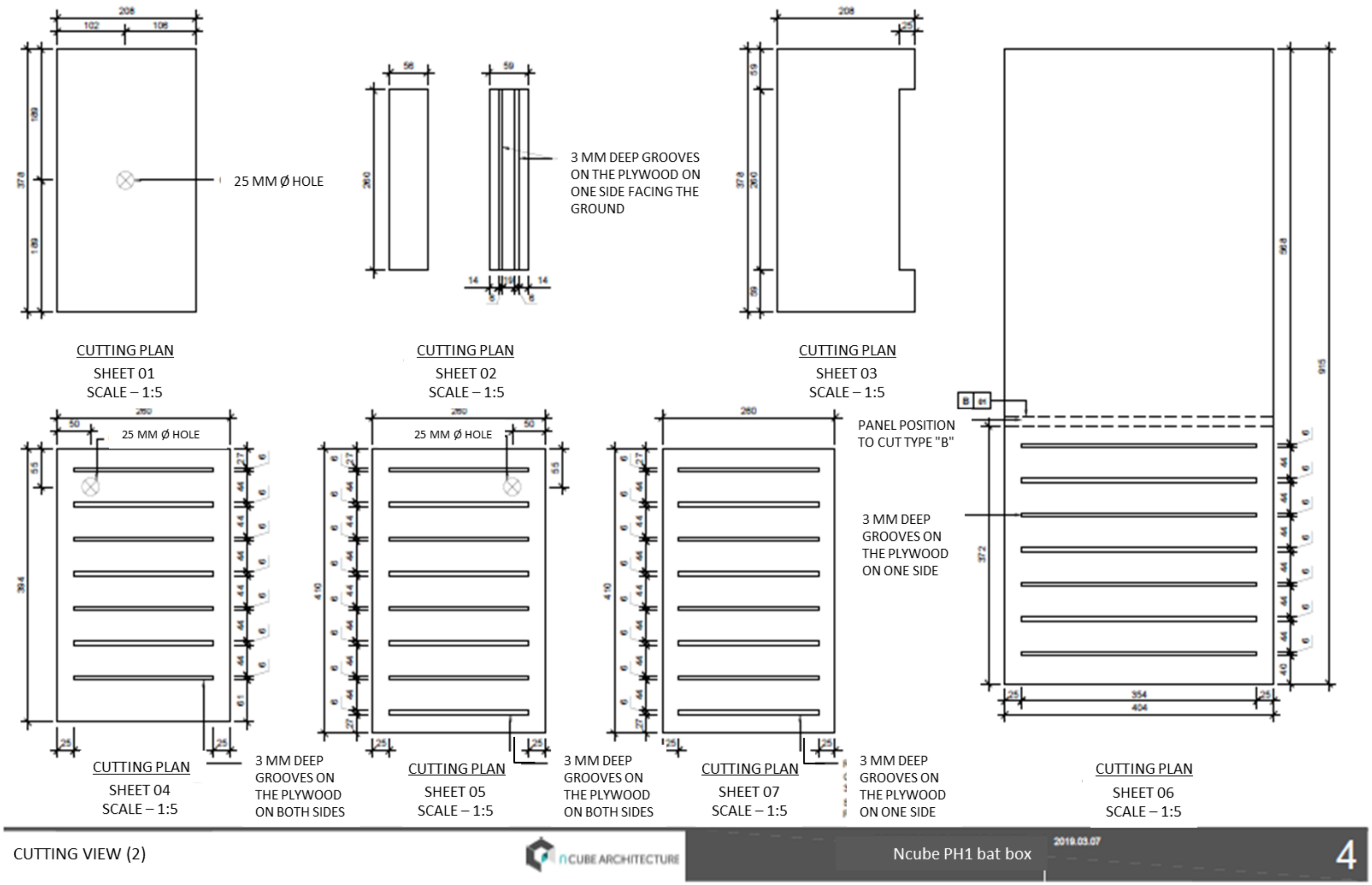

Figure S6. Design plan of the Ncube PH1 2019. Cutting plane view (2).

[1] *Email:* amelie.fontaine@mail.mcgill.ca
[2] *Current affiliation: Natural Resource Sciences, McGill University, 21,111 Lakeshore road, St-Anne-de-Bellevue, Qc, Can.*

Table S8. Site GPS position, location, climate, meteorological station type, and distance of the meteorological station from bat boxes.

Sites	GPS location	Regional location	Köppen climate type	Type	Distance
Warmer 1	45.50393, -75.81336	Outaouais	Humid continental	Davis vantage vue In situ	< 100 m.
Warmer 2	45.41109, -73.94468	Montreal	Humid continental	Environment Canada station (Ste Anne de Bellevue)	< 1000 m.
Intermediate 1	46.71640, -71.54246	Quebec	Humid continental	Davis vantage vue In situ	< 100 m.
Intermediate 2	46.59438, -72.11343	Quebec	Humid continental	Davis vantage vue In situ	< 100 m.
Intermediate 3	46.61753, -71.84768	Centre-du-Quebec	Humid continental	Davis vantage vue In situ	< 100 m.
Cooler 1	47.32049, -71.14918	Quebec	Humid continental	Environment Canada station (Forêt Montmorency)	< 500 m.
Cooler 2	51.68861, -75.82278	Nord-du-Quebec	Subarctic	Davis vantage vue In situ	< 100 m.

[1] *Email:* amelie.fontaine@mail.mcgill.ca
[2] *Current affiliation: Natural Resource Sciences, McGill University, 21,111 Lakeshore road, St-Anne-de-Bellevue, Qc, Can.*

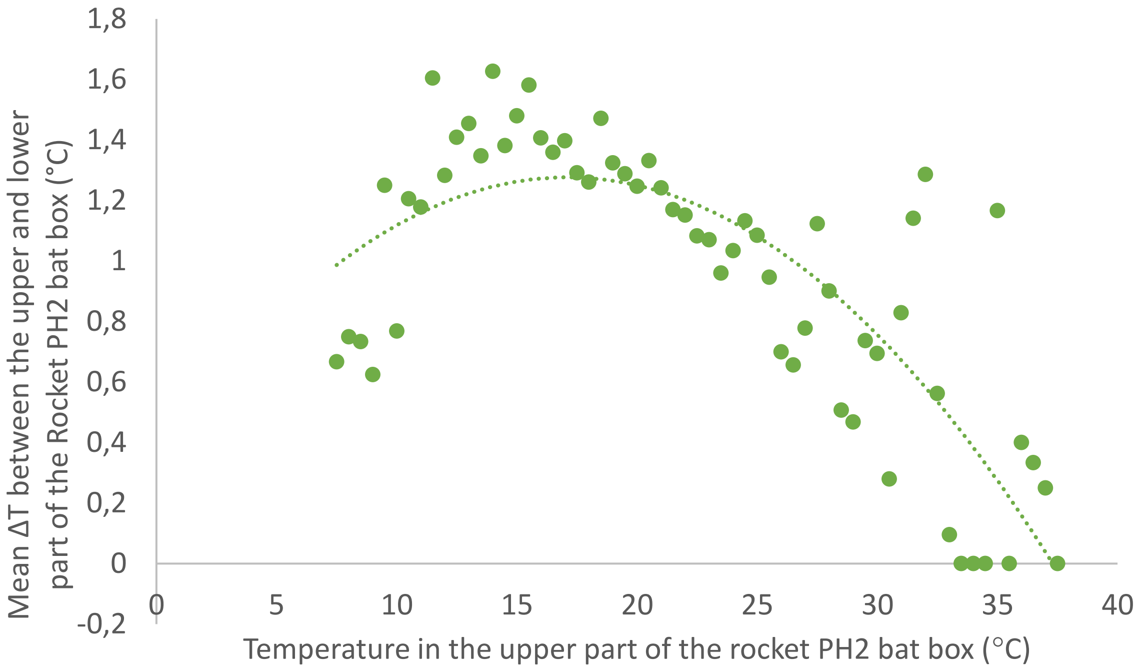

Figure S7. Temperature variation (°C) between the upper and lower part of the Rocket PH2 bat box on a pole from mid-May to Mid-September 2019 at site warmer 2 in Québec, Canada. The passive heating zones faced east and west. The green dotted line represents the trend curve following a polynomial function.

[1] *Email:* amelie.fontaine@mail.mcgill.ca
[2] *Current affiliation: Natural Resource Sciences, McGill University, 21,111 Lakeshore road, St-Anne-de-Bellevue, Qc, Can.*

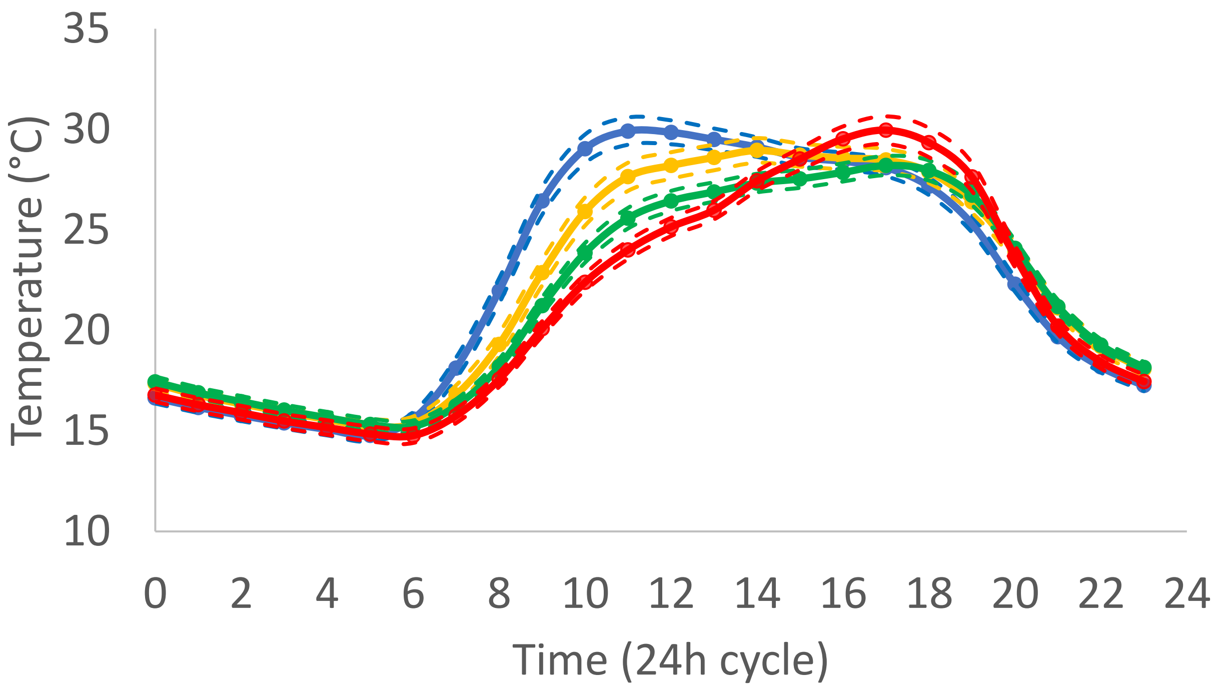

Figure S8. Daily mean temperature (°C) of classic 4-chambers bat boxes in the four chambers facing south from mid-May to Mid-September 2017 on poles at intermediate and warmer sites (n=2) Québec, Canada. C1 = chamber, C2 = chamber 2, C3 = chamber 3, C4 = chamber 4, from the front to the back of the bat box, and Text = external temperature.

[1] *Email:* amelie.fontaine@mail.mcgill.ca
[2] *Current affiliation: Natural Resource Sciences, McGill University, 21,111 Lakeshore road, St-Anne-de-Bellevue, Qc, Can.*

Table S9. GAMM model description for the orientation, mounting and model experiments.

**Treatments**	**Response variable (Y)**	**Explanatory variable (x)**	**Fixed factor**	**Random factor**
Orientation	T_{int}	Orientation	Time (as smoothed term and a categorical interaction with Orientation), T_{ext} (with a categorical interaction with Orientation), Date, Mounting	Location (with a random intercept), individual bat box identity (with a random intercept)
Mounting	T_{int}	Mounting	Time (as smoothed term and a categorical interaction with Mounting), T_{ext} (with a categorical interaction with Mounting), Date, Orientation	Location (with a random intercept), individual bat box identity (with a random intercept)
Model	T_{int}	Model	Time (as smoothed term and a categorical interaction with Model), T_{ext} (with a categorical interaction with Model), Week, Mounting	Location (with a random intercept), individual bat box identity (with a random intercept)

[1] *Email:* amelie.fontaine@mail.mcgill.ca
[2] *Current affiliation: Natural Resource Sciences, McGill University, 21,111 Lakeshore road, St-Anne-de-Bellevue, Qc, Can.*
